# Unlocking Mutual Gains—An Experimental Study on Collaborative Autonomous Driving in Urban Environment

**DOI:** 10.3390/s24010182

**Published:** 2023-12-28

**Authors:** Sumbal Malik, Manzoor Ahmed Khan, Hesham El-Sayed, Muhammad Jalal Khan

**Affiliations:** 1 College of Information Technology, United Arab Emirates University, Abu Dhabi 15551, United Arab Emirates; 201990107@uaeu.ac.ae (S.M.); 201990067@uaeu.ac.ae (M.J.K.); 2 Emirates Center for Mobility Research (ECMR), United Arab Emirates University, Abu Dhabi 15551, United Arab Emirates

**Keywords:** urban environment, collaborative driving, convoy, traffic flow, pollutant emission, coalitional game, incentivization

## Abstract

Convoy driving, a specialized form of collaborative autonomous driving, offers a promising solution to the multifaceted challenges that transportation systems face, including traffic congestion, pollutant emissions, and the coexistence of connected autonomous vehicles (CAVs) and human-driven vehicles on the road, resulting in mixed traffic flow. While extensive research has focused on the collective societal benefits of convoy driving, such as safety and comfort, one critical aspect that has been overlooked is the willingness of individual vehicles to participate in convoy formations. While the collective benefits are evident, individual vehicles may not readily embrace this paradigm shift without explicit tangible benefits and incentives to motivate them. Moreover, the objective of convoy driving is not solely to deliver societal benefits but also to provide incentives and reduce costs at the individual level. Therefore, this research bridges this gap by designing and modeling the societal benefits, including traffic flow optimization and pollutant emissions, and individual-level incentives necessary to promote convoy driving. We model a fundamental diagram of mixed traffic flow, considering various factors such as CAV penetration rates, coalition intensity, and coalition sizes to investigate their relationships and their impact on traffic flow. Furthermore, we model the collaborative convoy driving problem using the coalitional game framework and propose a novel utility function encompassing incentives like car insurance discounts, traffic fine reductions, and toll discounts to encourage vehicle participation in convoys. Our experimental findings emphasize the need to strike a balance between CAV penetration rate, coalition intensity, size, and speed to realize the benefits of convoy driving at both collective and individual levels. This research aims to align the interests of road authorities seeking sustainable transportation systems and individual vehicle owners desiring tangible benefits, envisioning a future where convoy driving becomes a mutually beneficial solution.

## 1. Introduction

The evolution of transportation systems is at a crossroads as we strive to address pressing issues such as traffic congestion, environmental pollution, and the coexistence of connected autonomous and human-driven vehicles (HDVs) on roadways. In this dynamic landscape, a specialized form of collaborative driving, referred to as convoy driving, has emerged as a promising solution to the multifaceted challenges facing modern transportation systems. Convoy driving is defined as the synchronized movement of more than one vehicle traveling in succession within a single lane with minimal spacing between them, all keeping a uniform speed and functioning as an interconnected convoy [1]. Convoy driving not only offers the potential to increase road capacity but also reduces pollutant emissions generated from transportation (note: from this point forward, the terms convoy and coalition are used interchangeably).

Recent studies indicate that the adoption rate of level 4 connected autonomous vehicles (CAVs) is projected to reach 24.8% by 2045 [2]. Another study suggests that CAVs will constitute approximately 75% of the vehicles on the road by 2050 [3]. However, the transition to fully upgraded CAVs will take time. Consequently, mixed traffic comprising both CAVs and HDVs is expected to coexist on the road for an extended period in the future. Existing research has demonstrated that in connected environments, CAVs exhibit enhanced information exchange and coalition-forming capabilities compared to conventional driving settings. The degree of clustering among CAVs referred to as coalition intensity, has a direct influence on the key performance indicators (KPIs) of mixed traffic capacity. While recent research has delved into the attributes of mixed traffic flow in recent years, there remains a need for further examination of the attributes of CAV convoys in mixed traffic settings, with a particular emphasis on coalition intensity and optimal coalition size.

The motivation of this study is to delve into the realization of convoy driving with the aim of uncovering its potential benefits within the context of mixed traffic flow. In this research, we investigate how convoy driving in mixed traffic can be leveraged to provide substantial societal advantages, such as traffic flow optimization and a reduction in pollutant emissions. The objective of convoy driving is not only to bring about societal benefits but also to incentivize and lower costs at the individual level [4]. However, one crucial aspect that has been overlooked in the literature regarding convoy driving is the willingness of individual vehicles to collaborate in coalition formations. While the collective societal benefits for traffic flow and environmental quality are evident, individual vehicles may not readily embrace this paradigm shift unless explicit tangible benefits and incentives are provided to motivate them. Therefore, an incentivization mechanism based on specific rules and policies is necessary, appealing to both authorities seeking to enhance the sustainability of transportation systems and individual vehicles seeking benefits for their participation. By aligning the interests of these two key stakeholders, we envision a future where convoy driving becomes a win-win solution—beneficial for society, the collective transportation system, and individual vehicle owners.

To the best of our knowledge, this is the first study that put efforts into investigating the benefits of coalition-driven travel for both stakeholders: governmental authorities and individual vehicles. Recently, researchers have shown significant attention to adopting game-theoretical learning solutions, specifically coalitional games [5], to address the challenges of collaborative driving. Considering the essence of this work, we opt for a coalitional game theory framework to design and model the collaborative convoy driving problem.

Some of the major contributions of this research are:

- Modeling the fundamental diagram of mixed traffic flow based on varying CAV penetration rates, coalition intensity, and sizes. This investigation explores key relationships and characteristics impacting traffic flow optimization.
- Investigating pollutant emissions of carbon dioxide (CO_2_) and nitrogen oxides (NO_x_) under different coalition settings.
- Modeling a coalitional game framework for the collaborative convoy driving problem. A unique multi-objective utility function incentivizes vehicles, offering diverse incentives and discounts, to form and operate within coalitions. The Shapley allocation is implemented ensuring a fair distribution of payoffs.
- Conducting numerical experiments to validate the practical advantages of the coalition-driven approach at both collective and individual vehicle levels.

The rest of the paper is organized into seven sections. Section 2 presents an analysis of related work and highlights the research gap. Section 3 is divided into two subsections that discuss the modeling of the fundamental diagram of mixed traffic flow and pollutant emission. Section 4 models the collaborative convoy driving problem and the utility function. Section 5 analyzes the proposed game using Shapley value. Section 6 provides a detailed discussion of the experimental settings and presents the results of a series of experiments. Finally, Section 7 summarizes the key findings and conclusions of the paper.

## 2. Related Work

This section is categorized into two subsections. Section 2.1 analyzes the related work concerning the societal benefits of convoy driving, while Section 2.2 discusses studies on the benefits and incentives of convoy driving at the individual level.

### 2.1. Societal Benefits of Convoy Driving

Research conducted by Yao et al. [6] studied how platoon management affects the flow of mixed traffic comprised of CAVs and HDVs. The fundamental diagram model and the stability of mixed traffic flow were explored by taking factors such as the size of the platoon and the CAV intensity. The fundamental diagram is a key concept in traffic flow theory which studies the connection between traffic flow, density, and speed on a roadway. A framework for the fundamental diagram of mixed traffic flow was established and theoretically demonstrated the relationship between traffic flow and the maximum size of the platoon. The findings indicate that larger platoon sizes can enhance traffic capacity but may negatively impact traffic flow stability; therefore, it is suggested to form platoons of sizes four to six vehicles. In another study, Yangsheng et al. [7] explored how the characteristics of CAV platoons influence the fundamental diagram in mixed traffic conditions. They devised platoon intensity for CAVs to gauge the degree of clustering among these vehicles. Subsequently, they introduced a traffic flow model leveraging the Markov chain model, aimed at determining the percentage of vehicles employing different car-following modes. Furthermore, they derived a fundamental diagram model considering the impact of both platoon intensity and platoon size. Various numerical experiments were undertaken to determine the effects of pertinent factors—specifically, the intensity of the platoon, platoon size, platoon management strategies, and CAV penetration rates on the fundamental diagram. Finally, simulation experiments were conducted to validate the theoretical model. The outcomes revealed that the intensity of the platoon, its size, and CAV penetration rates positively contribute to enhancing traffic capacity. Notably, a platoon size of two to four, with a consistent platoon intensity of 0.5, substantially boosts the capacity of traffic flow.

Yao et al. [8] analyzed mixed traffic flow comprising autonomous vehicles (AVs), CAVs, and HDVs with the maximum possible CAV platoon size. A probability distribution for the CAV platoon size using a Markov chain model was established. The numerical experiments demonstrated that as the penetration rate of CAVs increased, the probability of larger platoon sizes also increased. Conversely, when the maximum size of the platoon was predefined, the likelihood of specific platoon sizes occurring decreased. Furthermore, the theoretical experiments revealed that having a larger maximum platoon size enhanced traffic capacity but compromised the stability of mixed traffic flow. Additionally, an increase in the maximum size of the CAV platoon led to higher fuel consumption and emissions. However, the rate at which fuel consumption and emissions increased gradually diminished as the maximum platoon size of CAVs increased. Consequently, when the maximum platoon size surpassed the threshold platoon size, the fuel consumption and emissions showed no sensitivity to changes in the CAV platoon size. Li et al. [9] conducted research to explore how CAVs influence the attributes of the mixed traffic flow by introducing a fundamental diagram that factors in time delays, platooning intensity, and the deterioration of CAVs. Through a combination of sensitivity analysis and simulation results, their findings indicated that as the proportion of CAVs in the mixed traffic increased, both the maximum traffic flow and the optimal traffic density of mixed traffic flow experienced gradual increments. Furthermore, as platooning intensity gradually rose, the maximum traffic volume and optimal traffic density of mixed traffic flow also increased. Moreover, the free-flow speed had a favorable effect on the maximum traffic volume in mixed traffic flow. With a gradual increase in free-flow speed, the maximum traffic volume of mixed traffic flow likewise increased, while the critical density gradually decreased.

Ma et al. [10] designed a road capacity gain formula to model mixed traffic conditions based on the penetration rate of CAVs and various parameters. To analyze the interplay between HDVs and CAVs in mixed traffic scenarios, both the FVD model and the CAV model were employed. Through the simulations, it is explored how CAVs affect the capacity of the road and the pollutant emissions. The results suggested that once the platoon size exceeds 10, it has a minimal impact on road capacity. Moreover, regarding pollutant emissions, the incremental influence of CO_2_, particulate matter (PM), and NO_x_ decreases as the CAV penetration rate increases. In terms of CAV platoon size constraints, there is limited benefit in enlarging the platoon size when it surpasses 5 for CO_2_, volatile organic compounds (VOC), and NO_x_, but there is a substantial advantage for PM emissions. However, when the platoon size exceeds 15, the benefits of further enlargement become negligible. Therefore, a platoon size between 5 and 10 is optimal when considering both road capacity and pollutant emissions. Jiang et al. [11] introduced a definition and calculation formula for CAV platoon intensity. They focused on investigating how platoon intensity of connected and autonomous vehicles affected the fundamental diagram (volume-density relationship) and safety within mixed traffic flow. Through both theoretical and simulation assessment, the findings demonstrated that increased CAV penetration rates and increased platoon intensities both contribute to enhancing the traffic capacity of mixed traffic flow. This capacity increase is particularly pronounced when both the penetration rate and platoon intensity are at high levels, resulting in a 56% improvement in capacity compared to homogeneous traffic flow composed solely of HDVs. However, the limitation of this research is that they only investigated the impact of platooning intensity on traffic flow capacity and safety. In the future, further analysis could delve into how platoon intensity influences other aspects of mixed traffic flow, such as stability, fuel consumption, and pollutant emissions.

### 2.2. Benefits and Incentives of Convoy Driving at Individual Vehicle Level

Highway congestion often arises from the merging of vehicles, which is a complex maneuver due to factors such as traffic flow, driver behavior, and individual travel objectives. Hang et al. [12] proposed a coalitional game theory-based framework to address the challenge of multi-lane merging for CAV. The proposed framework incorporated two key components: a motion prediction module for predicting vehicle movements and a cost function that considers KPIs of safety, comfort, and effectiveness of the traffic. By combining a coalitional framework with model predictive control (MPC), it efficiently manages the decision-making process of connected autonomous vehicles in complex multi-lane merging scenarios. The results from two distinct case studies, encompassing the analyses of four different coalition types across various driving scenarios, provided compelling evidence of the rational decision-making capabilities of CAVs. Furthermore, the cost allocated to individual CAVs within the big coalition, computed using the Shapley value, was found to be less than that within a coalition of one player coalition underscoring the superior performance of the grand coalition in optimizing multi-lane merging for CAVs. In another research, Yang et al. [13] developed a decision-making approach for vehicle merging in multi-lane scenarios by integrating cooperative game theory with optimal control methods. They conducted experiments in a real-world road environment by simulating a two-lane mainline and a single-lane ramp. Within this setup, they defined two merging points on the mainline to address the optimization and sequencing challenges within the control zone. Furthermore, they formulated an objective optimization function that encompasses factors such as vehicle efficiency, fuel consumption, and driving comfort. The merging sequence was resolved using cooperative game theory, while the analytical solution for vehicle motion was derived through optimal control techniques.

Another research conducted by Hang et al. [14], formulated a collaborative decision-making framework for lane changes in autonomous vehicles. This framework employs a coalitional game theory by considering human-like driving characteristics, including traits such as aggression, moderation, and conservatism. The decision-making process was designed by a utility function comprised of three KPIs road safety, ride comfort, and efficiency of the traffic. Furthermore, the coalitional game was utilized to convert the lane-change decision-making scenario into an optimization problem. The results showed that the algorithm was capable of making lane-change decisions that were both safe and accurate for autonomous vehicles.

Effectively managing traffic flow at intersections, in urban environments, presents a considerable challenge. A study conducted by Hang et al. [15] introduced an approach aimed at mitigating the coordination and decision-making complexities encountered by CAVs when navigating unsignaled intersections. In order to tackle these challenges, they applied a Gaussian potential field approach to present an algorithm that evaluates risk by assessing the safety levels of nearby vehicles, effectively simplifying the decision-making process. The authors integrated considerations for both driving safety and the efficiency of overtaking maneuvers by CAVs to formulate a comprehensive decision-making cost function. Moreover, they outlined various decision-making limitations, encompassing control, traffic efficiency, ride comfort, and stability factors. Utilizing this cost function and the defined constraints, they devised two distinct fuzzy coalitional game techniques: single-player coalition and grand coalition, addressing the decision-making challenges faced by CAVs at unsignaled junctions. The results state that the suggested approach allowed CAVs to make safe and rational decisions.

Little work [16,17,18] has also been conducted on incentivization mechanisms such as monetary rewards and reputation. However, the focus has been on incentivizing the vehicles to become the leader of the convoy, rather than motivating them to form and travel in a convoy.

A comparison of the relevant research, examining various criteria, is depicted in Table 1. In what follows next, we highlight the limitations of the current literature: (i) most studies have a singular focus, either on the societal aspect or on the individual benefits, with none addressing both perspectives simultaneously; (ii) research on the fundamental diagrams of relationships, such as speed–density and speed–volume, remains relatively shallow; (iii) none of the studies explored the impact of different free-flow speeds and minimum safe distance on the fundamental diagram; (iv) notably, only two studies, as indicated in Table 1, delve into the effects of CAV on CO_2_ and NO_x_ emissions; (v) most studies predominantly focus on modeling the utility function based on just one or two KPIs, without considering any incentivization mechanisms.

## 3. Modeling of the Societal Benefits

This section is structured into two sub-sections. Section 3.1 focuses on the modeling of mixed traffic flow, while Section 3.2 discusses the modeling of pollutant emissions.

### 3.1. Mixed Traffic Flow Modeling

In this section, we model the fundamental diagram and analyze the mixed traffic flow by investigating the attributes of traffic flow and various relationships, including density–flow, speed–flow, and speed–density. The modeling is based on several important factors, such as coalition size, coalition intensity, CAV penetration rate, and free-flow speed. It is essential to emphasize that only the car-following behavior of the vehicles is modeled and not the lateral behavior such as lane change. In this study, we establish the definition of the term coalition as follows:

**Definition 1.**  *A coalition, denoted as C, is characterized by a set of vehicles represented by N, where each vehicle is indexed from 1 to n. All vehicles within the coalition move at a uniform speed of V, maintaining a spacing of $T_{m}$, while the coalition’s size, CS, is constrained to be no greater than ${\mathrm{CS}}_{max}$.*

#### 3.1.1. Spatial Arrangement of Vehicles in Mixed Traffic

**Figure 1:**
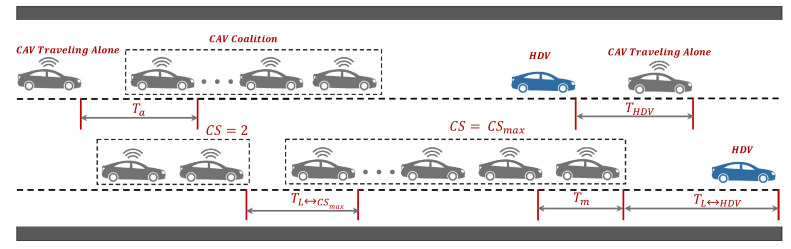
Types of headways of spatially distributed vehicles in the mixed traffic flow.

We assume that the mixed traffic is comprised of both CAVs and HDVs which are randomly spatially distributed on the road; therefore, there are five types of headway between the vehicles that we consider in this study illustrated in Figure 1. (i) $T_{HDV}$: the headway spacing between a trailing HDV and the leading HDV or CAV; (ii) $T_{a}$: the spacing between the CAVs traveling alone without the member of any coalition; (iii) $T_{L↔HDV}$: the gap between a leader of a CAV coalition and a preceding HDV; (iv) $T_{L↔{\mathrm{CS}}_{max}}$: the space between the leader of a CAV coalition and the leading maximum size coalition denoted as ${\mathrm{CS}}_{max}$. If a coalition’s size is smaller than ${\mathrm{CS}}_{max}$, then the subsequent vehicle after the last one in this coalition is HDV; (v) $T_{m}$: the spacing between the coalition members.

Given that HDVs lack communication with their preceding vehicles, we posit that $T_{HDV}$ is greater than the other four headway, meaning thereby $T_{HDV}=\max\{T_{HDV},$$T_{a},T_{L↔HDV},T_{L↔{\mathrm{CS}}_{max}},T_{m}\}$. In contrast, CAVs within the same coalition maintain more frequent communication and access real-time data, leading us to set $T_{m}$ at $T_{m}=\min\{T_{HDV},$$T_{a},T_{L↔HDV},T_{L↔{\mathrm{CS}}_{max}},T_{m}\}$. Both $T_{a}$ and $T_{L↔HDV}$ share similar attributes, involving one-way communication and following HDVs, which naturally make them smaller than $T_{HDV}$. Additionally, $T_{L↔HDV}$ is greater than $T_{L↔{\mathrm{CS}}_{max}}$. However, it is important to note that $T_{L↔HDV}$ exceeds or equal to $T_{a}$ as the leader of the CAV coalition is responsible for ensuring the safety of all coalition members.

#### 3.1.2. Coalition Intensity

In the mixed traffic flow, the rate at which the CAVs are spatially distributed on the road plays a crucial role in determining their varying degrees of clustering, strength referred to as coalition intensity. It is measured as the proportion of the actual count of CAVs involved in a coalition to the overall count of CAVs within the mixed traffic. A ratio of 1 indicates that all CAVs within the mixed traffic can create coalitions with sizes no larger than the maximum permissible. Conversely, when this ratio approaches its minimum value, it signifies that CAVs within the mixed traffic are inclined to travel alone separated by the HDVs, resulting in minimal coalition intensity. The formulation of coalition intensity is presented in Equation (1).

(1) $$\mathrm{CI}=\frac{\sum_{\mathrm{CS}=2}^{{\mathrm{CS}}_{max}}\;\mathrm{CS}\times \;M_{\mathrm{CS}}}{N_{v}\times P_{r}}$$

**Figure 2:**
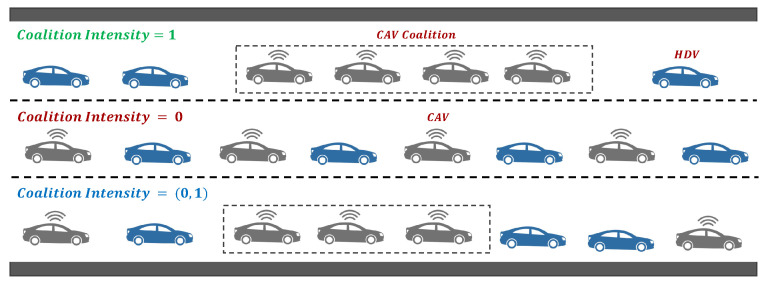
Levels of coalition intensity.

In Equation (1), the CI is the intensity of forming a coalition of CAVs; CS is the size of the coalition, ${\mathrm{CS}}_{max}$ is the maximum size of the coalition; $M_{\mathrm{CS}}$ is the number of coalitions of size CS; $N_{v}$ is the count of total vehicles on the road comprised of connected autonomous and human-driven vehicles; and the $P_{r}$ is the market penetration rate of connected autonomous vehicles. We can say that the value of CI lies from $\left[max\left(\;0\;,\;2P_{r}-\frac{1}{P_{r}}\right),1\right]$ and when the $P_{r}\leq 0.5$ the value of CI is between (0,1) and when the $P_{r}>0.5$ the CI is $\left[2P_{r}-\frac{1}{P_{r}},1\right]$. Given the $P_{r}=0.5$, the CI can be classified into three main levels as presented in Figure 2.

(i) CI=1 when $P_{r}=1$ meaning thereby that all the CAVs on the road can form coalitions; (ii) CI=0, when the $\mathrm{CI}=max\left\{0\;\;,2P_{r}-\frac{1}{P_{r}}\right\}$ it represents that no CAVs would travel in the coalition and they follow the HDVs; (iii) CI=(0,1), when $\max\left\{0\;,\;2P_{r}-\frac{1}{P_{r}}\right\}$<CI<1 shows that the some of the CAVs are traveling alone. Moreover, for any given level of CI the maximum penetration rate $P_{r}^{max}$ of CAVs is computed using Equation (2).

(2) $$P_{r}^{max}=\frac{1}{2-\mathrm{CI}}$$

#### 3.1.3. Probability Distribution of Car-Following Modes

For the car-following modes discussed in Section 3.1.1, the probability distributions are formulated by considering both the coalition intensity CI and the CAV penetration rate $P_{r}$. Let $p_{HDV}$, $p_{a}$, $p_{L↔HDV}$, $p_{L↔{\mathrm{CS}}_{max}}$, and $p_{m}$ denote the probability distribution of $T_{HDV}$, $T_{a}$, $T_{L↔HDV}$, $T_{L↔{\mathrm{CS}}_{max}}$, and $T_{m}$, respectively. Therefore, the probability distribution can be computed as follows:

- The spatial probability distribution of an HDV is computed as in Equation (3) where $P_{r}$ is the market penetration rate of connected autonomous vehicles.
  (3) $$p_{HDV}=1-P_{r}$$
- The probability of the CAV traveling alone and not being a member of any coalition is calculated as in Equation (4) where the CI represents the intensity of the coalition formation.
  (4) $$p_{a}=\left(1-\mathrm{CI}\right)\times P_{r}$$
- The scenario where the coalition leader follows an HDV requires two conditions to be met at the same time: (i) it must follow a human-driven vehicle, and (ii) it should be traveling in a coalition. The probability distribution can be calculated as in Equation (5).
  (5) $$p_{L↔HDV}=\mathrm{CI}\times P_{r}\times \left(1-P_{r}\right)$$
- The scenario where the coalition leader follows a maximum size coalition ${\mathrm{CS}}_{max}$ should meet two conditions such as it should have a n≥1 consecutive CAV coalitions ahead of it, as a new coalition is established when the count of CAVs exceeds the ${\mathrm{CS}}_{max}$, and the vehicle behind it is a CAV. Consequently, the probability of this scenario is computed as follows in Equation (6).
  (6) $$p_{L↔{\mathrm{CS}}_{max}}=\left(1-P_{r}\right)\times P_{r}\times \mathrm{CI}\times \frac{P_{r}^{{\mathrm{CS}}_{max}}}{1-P_{r}^{{\mathrm{CS}}_{max}}}$$
  The combined probability distribution of both CAV leaders can be computed by combining Equations (5) and (6) as in Equation (7).
  (7) $$p_{L}=\left(1-P_{r}\right)\times P_{r}\times \mathrm{CI}\times \frac{1}{1-P_{r}^{{\mathrm{CS}}_{max}}}$$
- Assuming that the follower *i* of a coalition C is traveling at a *j*th position in the coalition where $j\in [2,CS_{max}]$, the probability distribution of this mode is calculated as the sum of the *i* follower within the coalition, as shown in Equation (8).
  (8) $$p_{m}=\frac{P_{r}\times \mathrm{CI}\times \left(P_{r}-P_{r}^{CS_{max}}\right)}{1-P_{r}^{CS_{max}}}$$

**Figure 3:**
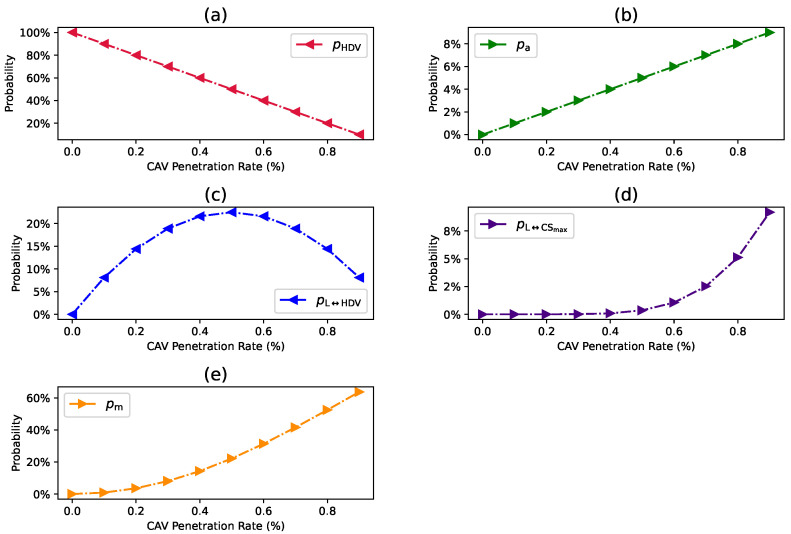
Probability distribution of CAVs headway.

Using Equations (3)–(6), and (8) the probability of CAV headways is calculated by setting CI=0.9 and CS=6. As depicted in Figure 3, the changes in the probability of vehicle headway are associated with variations in $P_{r}$ of CAVs. This illustration demonstrates the feasibility of computing the probability distribution of headway considering the CI and CS. It is evident from Figure 3a that the probability of $p_{HDV}$ exhibits a linear decrease as $P_{r}$ gradually increases. This decrease arises from the reduction in the proportion of HDVs as $P_{r}$ accumulates over time. In (b), the probability of $p_{a}$ steadily increases with the rise in $P_{r}$. In (c), the probability of $p_{L↔HDV}$ first increases and then decreases with the increase of $P_{r}$. The decrease is due to the reduction in the proportion of HDVs as the CAV penetration rate increases. In (d), the probability of $p_{L↔{\mathrm{CS}}_{max}}$ increases very slowly as the $P_{r}$ increases. Notably, in (e), the probability of $p_{m}$ undergoes an exponential surge as $P_{r}$ gradually increases. This phenomenon is driven by the escalating number of coalitions with the incremental growth of $P_{r}$.

#### 3.1.4. Car-following Models for Mixed Traffic Flow

This section provides a brief overview of the selected car-following models, which are derived from the car-following behaviors of various vehicle categories presented in Section 3.1.1.

- *Intelligent Driver Model*—The Intelligent Driver Model (IDM) finds widespread application in the analysis of traffic flow characteristics [23]. With a concise set of parameters that hold clear physical interpretations in line with a driver’s real-world driving familiarity, this model effectively captures the car-following behavior of HDVs. Therefore, this research leverages the IDM to model the behavior of HDVs as shown in Equations (9) and (10).
  (9) $$a\left(t\right)=\alpha \left[1-{\left(\frac{V_{n}\left(t\right)}{V_{f}}\right)}^{4}-{\left(\frac{S(t)}{H_{n}\left(t\right)-L_{v}}\right)}^{2}\right],$$
  (10) $$S\left(t\right)=S_{min}+max\left(0,V_{n}\left(t\right)\times T_{HDV}+\frac{V_{n}\left(t\right)\times \left(V_{n}\left(t\right)-V_{n-1}\left(t\right)\right)}{2\sqrt{\beta \gamma }}\right)$$
  where a(t) is the instantaneous acceleration of the vehicle *n* at time *t*; β and γ represents the desired maximum acceleration and comfortable deceleration, respectively, $V_{n}\left(t\right)$ represents the velocity of vehicle *n* during the time *t*.; $V_{f}$ denotes the free-flow speed; $S_{min}$ represents the minimal stopping distance; S(t) is the desired spacing; $H_{n}\left(t\right)$ denotes the real gap between vehicle *n* and the vehicle in front, which is n−1; $L_{v}$ is the vehicle length; and $T_{HDV}$ is the desired headway.
- *Cooperative Adaptive Cruise Control*—When connected and autonomous vehicles travel in collaborative convoys, the vehicles engage in cooperative adaptive cruise control mode, facilitated by V2V communication capabilities which allows the vehicles to travel with shorter spacings between the adjacent vehicles. The CACC model [24] is utilized to depict the car-following behaviors of CAVs within the convoys. The formulae of this method are discussed in Equations (10) and (12).
  (11) $$V_{n}\left(t+\Delta t\right)=V_{n}+K_{p}e_{n}\left(t\right)+K_{d}{\dot{e}}_{n(t)}$$
  (12) $$e_{n}\left(t\right)=H_{n}\left(t\right)-L_{v}-S_{min}-T_{m}\times V_{n}\left(t\right)$$
  where Δt represents the time interval; $K_{p}$ and $K_{d}$ denote the control gains; $e_{n}\left(t\right)$ represents the deviation in spacing between the actual and intended spacings of vehicle *n* at time *t*; $T_{m}$ denotes the targeted following distance between the CACC-equipped vehicles.
- *Adaptive Cruise Control*—The adaptive cruise control (ACC) model [25] developed by the PATH’s laboratory is employed to simulate the car-following behaviors of both individual CAVs traveling alone and the leading CAVs in convoy formations. The mathematical formula to model the ACC is presented in Equation (13).
  (13) $$a_{n}\left(t\right)=K_{1}\left(H_{n}\left(t\right)-L_{v}-S_{min}-T_{*}\times V_{n}\left(t\right)+K_{2}\left(V_{n-1}\left(t\right)-V_{n}\left(t\right)\right)\right)$$
  here, $K_{1}$ represents the control gain related to the spacing error between vehicles; $K_{2}$ is the control gain associated with speed differences; $T_{*}=T_{a}$ signifies the desired time headway for individual CAVs; $T_{*}=T_{L↔HDV}$ is the headway between the coalition leader following the HDVs, and $T_{*}=T_{L↔{\mathrm{CS}}_{max}}$ is the headway between the coalition leader following the maximum size coalition.

#### 3.1.5. The Fundamental Diagram Model—Mixed Traffic Flow

The fundamental diagram of mixed traffic discusses the relationship among three pivotal factors (such as traffic flow, density, and speed) that characterize the flow of traffic at an equilibrium state. In this state, every vehicle’s speed in the traffic stream equals the equilibrium speed, $V_{e}$, while the acceleration of all vehicles becomes zero. The equilibrium conditions of three models, discussed in Section 3.1.4 are presented below:

(14) $$V_{n}\left(t\right)=V_{e}$$

(15) $$V_{n}\left(t\right)=V_{n-1}\left(t\right)$$

(16) $${\dot{V}}_{n}\left(t\right)=0$$

In mixed traffic flow, the headway between vehicles in the equilibrium state is defined by their respective car-following models. As a result, the equilibrium spacings for these models are derived by substituting Equations (14)–(16) into Equations (9)–(13) as presented in Table 2.

Considering the probability of headway of different models, we derive the average headway $\hat{H}$ for mixed traffic flow using Equation (17):

(17) $$\hat{H}=p_{HDV}\times H_{HDV}+p_{a}\times H_{a}+p_{L}\times H_{L↔HDV}+p_{L}\times H_{L↔{\mathrm{CS}}_{max}}+p_{m}\times H_{m}$$

To formulate the general derivation framework, the mixed traffic flow’s density-speed fundamental diagram model is derived by leveraging the definition of traffic density as presented in Equation (18).

(18) $$K=\frac{1}{\hat{H}}=\frac{1}{p_{HDV}\times H_{HDV}+p_{a}\times H_{a}+p_{L}\times H_{L↔HDV}+p_{L}\times H_{L↔{\mathrm{CS}}_{max}}+p_{m}\times H_{m}}$$

where K is the traffic density measured in vehicles per kilometer per lane (Veh/km/ln) and $\hat{H}$ is the average headway spacing.

In accordance with the relationship between traffic volume, speed, and density, we derive the volume-density fundamental model for mixed traffic, which takes into account both coalition intensity and coalition size. This model is expressed in Equation (19).

(19) $$\left\{\begin{array}{cc} K=\frac{1}{a+b+c+d+e} & \\ Q=K\times V_{e} \end{array}\right.$$

here in Equation (19):

(20) $$a=\left(1-P_{r}\right)\left(\frac{S_{\min}+V_{e}\times T_{HDV}}{\sqrt{1-{\left(\frac{V_{e}}{V_{f}}\right)}^{4}}}+L_{v}\right)$$

(21) $$b=P_{r}\times \left(1-\mathrm{CI}\right)\times \left(L_{v}+S_{\min}+V_{e}\times T_{a}\right)$$

(22) $$c=\left(1-P_{r}\right)\times P_{r}\times \mathrm{CI}\times \left(L_{v}+S_{\min}+V_{e}\times T_{L↔HDV}\right)$$

(23) $$d=\left(1-P_{r}\right)\times P_{r}\times \mathrm{CI}\times \frac{P_{r}^{{\mathrm{CS}}_{\max}}}{1-P_{r}^{{\mathrm{CS}}_{\max}}}\times \left(L_{v}+S_{\min}+V_{e}\times T_{L↔{\mathrm{CS}}_{max}}\right)$$

(24) $$e=P_{r}\times \mathrm{CI}\times \frac{P_{r}-P_{r}^{{\mathrm{CS}}_{\max}}}{1-P_{r}^{{\mathrm{CS}}_{\max}}}\times \left(L_{v}+S_{\min}+V_{e}\times T_{m}\right)$$

and Q is the traffic volume of the mixed traffic measured in vehicles per hour per lane (veh/h/ln).

### 3.2. Pollutant Emission Modeling

In this research, we investigate the influence of collaborative convoy driving on the different pollutant emissions $P_{e}$ such as carbon dioxide (CO_2_) and Nitrogen Oxides (NO_x_) generated by the vehicles. Several traffic emission models have been explored in existing literature, and one of the most widely employed models is the VT-Micro model.

The VT-Micro model, introduced by Ahn et al. [26] was fine-tuned using empirical data. This model estimates emissions and fuel consumption based on the instantaneous speed *V* and instantaneous acceleration *a* of the vehicle V. Due to its simplicity and widespread use in transportation research for computing fuel consumption and transportation emissions, we adopt the VT-Micro model for this study to calculate emissions for mixed traffic flows. The model is formulated as below:

(25) $$\ln\left(MOE_{e}\right)=\left\{\begin{array}{cc} \sum_{i=0}^{3}\sum_{j=0}^{3}\;\left(R_{i,j}^{e}\;\times \;V^{i}\;\times \;a^{j}\right) & \;\;for\;a(t)\geq 0\\ \sum_{i=0}^{3}\sum_{j=0}^{3}\;\left(S_{i,j}^{e}\;\times \;V^{i}\;\times \;a^{j}\right) & \;\;for\;a(t)<0 \end{array}\right.$$

The model presented in Equation (25) is discussed as below:

ln: is the natural logarithm function of a real number.
$MOE_{e}$: represents the rate of instantaneous fuel consumption or emission measured in liter per kilometer (L/km) or grams per second (g/s), respectively.
*e*: it represents an indicator for the type of fuel consumption or emissions such as CO_2_ and NO_x_ emissions. It is important to note that *e* is not an exponential function.
V: is the instantaneous speed of the CAV in km/h.
*a*: is the instantaneous acceleration of the CAV measured in km/h/s.
$R_{i,j}^{e}$: represents the regression coefficient in the model for $MOE_{e}$ at speed exponent *i* and acceleration exponent *j* for positive accelerations.
$S_{i,j}^{e}$: represents the regression coefficient in the model for $MOE_{e}$ at speed exponent *i* and acceleration exponent *j* for negative accelerations.

Moreover, it is seen from Equation (25) that the model deals separately with the positive and negative acceleration of the vehicles. We utilize Equation (25) with different regression coefficients to compute the transportation emissions of CO_2_ and NO_x_ generated by CAVs. The VT-Micro coefficients to estimate the CO_2_ and NO_x_ are presented in Appendix A, Table A1 and Table A2, respectively.

## 4. **Collaborative Convoy Driving—A Coalitional Game**

In this section, we delve into the modeling of collaborative convoy driving, framing it as a coalition game. We design the underlying principles and the utility function to represent the problem.

### 4.1. System Modelling

We characterize a setup within a specific geographical area denoted as R, encompassing a road segment spanning K kilometers. This setup comprises connected autonomous vehicles (CAVs) denoted by N, where the value of *n* represents the total count of CAVs in the system and can be expressed as 2, $2^{2}$, or $2^{3}$. These CAVs are equipped with advanced communication technologies that enable them to establish connections, cooperate, and coordinate with nearby vehicles, referred to as NV, within a specified distance range. During any given moment in time, denoted as *t*, a subset of CAVs, represented as S(t), have the capability to engage in collaborative driving and form coalitions, termed C. These coalitions are established with NV traveling in the same direction along the same route *R* with the objective of optimizing the traffic flow, improving safety, and reducing pollutant emissions, as well as providing individual benefits.

A vehicle V is not required to stay in a coalition C for each time step. Each CAV V from the set N can operate in either of two distinct travel modes: traveling alone $T_{a}$ or traveling in a coalition $T_{C}$. The selection of the travel mode for V is made by considering which option offers the greatest benefit. Additionally, for vehicle V to participate in a coalition, it must meet and agree to specific conditions, including parameters such as speed V and inter-vehicle distance $T_{m}$, among others. It is important to note that the size of these coalitions is limited by a threshold denoted as ${\mathrm{CS}}_{max}$. This constraint is of paramount importance for upholding stability and efficiency in the intricate urban setting. Additionally, we model the assumption that at any given time *t*, V can only be a member of one coalition, as denoted by C(j)∩C(k)=∅,∀j,k∈N. However, several key questions arise in this context: (i) When is it advantageous for V to join a coalition? (ii) How long should a vehicle remain in a coalition? (iii) What is the optimal approach for creating these coalitions and fairly distributing the benefits among coalition members?

Considering the collaborative essence of our research problem, we employ an approach that aligns best with our objectives: the coalitional game theory (CGT) framework. We use this approach to represent collaborative driving as a coalition formation problem. The formulation of coalitional game theory is outlined below.

**Definition 2.**  *A coalition game, symbolized as G, is represented by a pair 〈N,ν〉, N consists of the integers from 1 to n signifies a finite group of players seeking to create coalitions C, with $C\in 2^{N}$. When C comprises a single player, it is referred to as an individual-player coalition, whereas a coalition encompassing each player is termed a big coalition. The ν is a function that maps to real values known as the characteristic function and represented as $\nu :2^{N}\to R$. It assigns a payoff, denoted as ν(C), to each coalition C⊆N.*

In the realm of urban convoy driving, we utilize the coalitional game framework to represent how vehicles collaborate to create convoys, aiming to reap the advantages of coalition-driven travel. In the subsequent discussion, we delve into the elements of this coalitional game framework and discuss their significance within this context.

- *Players*: it refers to the vehicles operating within the urban settings that strive to achieve their specific goals, such as reducing the consumption of fuel consumption, minimizing traveling time, and obtaining other discounts.
- *Coalition*: is referred to as a collection of vehicles that create convoys. It is defined as vehicles moving closely together in a synchronized fashion.
- *Characteristic Function*: it is the function represented as ν which assigns a value to every coalition. In the context of collaborative driving, ν encapsulates the benefits and compromises associated with coordination in addition to the restrictions placed on the vehicles. The function ν acts as a utility function, with every player or coalition striving to optimize its value. A thorough discussion on the modeling of the suggested utility function is available in Section 4.3. When considering a coalition C, the utility U is determined by aggregating the individual utilities of all vehicles of that coalition. The formation of coalitions provides vehicles with the advantages of fuel savings, reduced travel times, and additional discounts on car insurance, traffic fines, and tolls. Vehicles may come across multiple coalitions, and each vehicle V chooses the optimal coalition by maximizing its utility. Given the dynamic environment members of the coalition, represented as V∈C, have the liberty to exit the coalition whenever they choose while following the established rules and regulations. Nevertheless, vehicles typically remain within their current coalitions until a more favorable option emerges or until their routes align.

### 4.2. Underlying Assumptions

This study delves into several key assumptions, which are outlined as follows:

- The size of the coalition C should be greater than 2 and less or equal to the maximum size of the coalition $2<|\mathrm{CS}|\leq {\mathrm{CS}}_{max}$.
- The utility U of a player is exclusively determined by the members of the C indicating that external factors have no impact on it.
- The suggested algorithm runs across all vehicles, helping them in determining whether to participate in the coalition.

### 4.3. Proposed Utility Function for Autonomous Vehicle Coalitions

Within this section, we formulate a unique multi-objective utility function for the connected autonomous vehicle V as it travels within a coalition C. The proposed multi-criteria function accommodates various components to determine the utility of V desiring to join the coalition C. Let $U_{i,C}$ be the utility of vehicle *i* when traveling in a coalition C is formulated as below:

(26) $$U_{i,C}:=\left[\underset{\mathrm{Collaboration}\mathrm{Window}}{\underset{︸}{\left(\rho \times C_{W}\right)}}\;\times \;\;\underset{\mathrm{Objective}}{\underset{︸}{e^{Gain}}}\right]-\underset{\mathrm{Cost}}{\underset{︸}{J_{C}}}$$

The proposed utility function presented in Equation (26) is decomposed into three main components namely: (i) the collaboration window function which computes the time during which a vehicle V intends to operate within the coalition C; (ii) objective function models advantages that a V experiences while journeying within C and the (iii) the cost function computes the expenses that a V might potentially face when joining the C.

It is important to note that the proposed function consolidates all the criteria into a unified metric to evaluate the advantages of traveling in coalition considering the realistic characteristics of each parameter. The normalization is employed due to the varying units of measurement for the parameters and the normalization values range within the interval of [1,5]. Subsequently, we will delve into each component comprehensively:

#### 4.3.1. Collaboration Window Function

The Collaboration Window function, represented as $C_{W}$, is crafted to determine the timeframe during which a vehicle V indicates its willingness to be a member of a coalition C. The function to calculate the $C_{W}$ is presented below:

(27) $$C_{W}=\alpha_{1}\times \;C_{E}\;+\;\alpha_{2}\times \;T_{d}\;+\;\alpha_{3}\times \;O_{V↔C}^{D}\;+\;\alpha_{4}\times \;\Delta V_{dif}$$

In Equation (27), $C_{W}$ takes into account four critical factors: (i) the complexity of the environment $C_{E}$ in which the vehicle V operates; (ii) the overlapping distance $O_{V↔C}^{D}$ between V and the coalition C; (iii) the estimated time $T_{d}$ required for V to arrive its destination *d* when traveling solely; and (iv) $\Delta V_{dif}$ computes the speed differential between V desired speed and the speed provided within the coalition C. The weighting coefficients $\alpha_{1}$, $\alpha_{2}$, $\alpha_{3}$, and $\alpha_{4}$ reflect the influence of each factor, and their total add upto 1 as expressed by $\sum_{i=1}^{n}\left(\alpha_{i}\right)=1$.

We believe, that computing the optimized values of $C_{W}$ is a pivotal element in forming coalitions of CAVs which benefits the vehicles with the enhanced safety, efficiency, and convenience of collaborative driving. The optimal $C_{W}$ value for V varies based on the surrounding environment. In complex settings like roundabouts, shorter $C_{W}$ values help navigate safely. This enhances collaborative driving by increasing awareness and facilitating safer traversal of roundabouts. Conversely, on highways, longer-lasting coalitions are possible due to stable conditions, enabling safety and efficiency optimization through collaboration.

In the subsequent sections, we delve into each element of $C_{W}$ in detail:

- ***Environmental Complexity—*** The complexity of the environment for vehicle V refers to the degree of the dynamicity of its driving environment. This encompasses variables like traffic patterns, weather conditions, pedestrians and cyclists, the presence of other vehicles, and infrastructure components like traffic signals and signs. The environment in which V travels, especially at intersections, junctions, and other high-traffic zones, plays a pivotal role in the vehicle’s decision of whether to form and travel in a coalition C. Such road segments frequently demand precise coordination and communication among multiple vehicles to ensure secure and efficient navigation. As a result, V should be capable of assessing the $C_{E}$ within its immediate vicinity to make an optimal decision regarding coalition formation.
  The traffic environment encompasses various external factors that affect autonomous driving such as weather conditions, road conditions, and the behavior of other road participants. We assert that the $C_{E}$ is an objective characteristic that mainly depends on the physical attributes of the environmental elements such as the static element complexity $S_{ec}$ and the dynamic element complexity $D_{ec}$. The $S_{ec}$ comprises stationary elements of the environment, such as road segments, tunnels, signage, road markings, vegetation, etc., whereas the $D_{ec}$ encompasses dynamic elements such as vulnerable road users, vehicles, and non-motorized vehicles.
  To calculate the $C_{E}$ for V, we adopt the equation from the study conducted by Cheng et al. [27]. This equation combines the complexity of both static and dynamic elements, and the result gives the overall complexity of the traffic environment. The formula is presented in Equation (28), where the values of the weighing coefficients $\lambda_{1}$ and $\lambda_{2}$ have been set to 0.35 and 0.65, respectively.
  (28) $$C_{E}=\lambda_{1}\times \;S_{ec}+\lambda_{2}\times \;D_{ec}$$
  Moreover, utilizing the values obtained for $C_{E}$ from Equation (28), we classify the $C_{E}$ for V into different levels, as presented in Equation (29).
  (29) $$C_{E}=\left\{\begin{array}{cc} 0.8\leq C_{E}\leq 1 & \;\;\mathrm{Extremely}\;\mathrm{Complex}\\ 0.6\leq C_{E}<0.8 & \;\;\mathrm{More}\;\mathrm{Complex}\\ 0.4\leq C_{E}<0.6 & \;\;\mathrm{Average}\\ 0<C_{E}<0.4 & \;\;\mathrm{Simple} \end{array}\right.$$
- ***Expected Time to Reach Destination—*** The expected time for a vehicle to arrive at its destination *d* in time *t* represents the time it will take for V to journey from its present location $P_{v}\left(u,v\right)$ to the designated destination. This estimation takes into account various factors, including distance, speed, and potential stops or delays during the journey. The travel time $T_{d}$ for V to arrive at its *d* when traveling independently is calculated as below:
  (30) $$T_{d}=\frac{D}{V}\;+\left(N_{s}^{v}\;\times \;S_{t}^{v}\right)$$
  In Equation (30), *D* represents the distance that V must traverse to reach the destination *d*, with a traveling speed denoted as V. $N_{s}^{v}$ represents the count for potential stops that V could encounter on its journey to *d* because of congestion, and $S_{t}^{v}$ represents the duration of time spent at each stop. Calculating $T_{d}$ plays a pivotal role for CAV V in determining whether to establish a coalition. For instance, if V calculates its $T_{d}$ and concludes that it will arrive at its destination *d* in a relatively brief timeframe, it may not perceive it as beneficial to become part of a coalition C. This is due to the fact that the potential time savings achieved by forming C may not be sufficient to compensate for the increased expenses associated with collaborating with $N_{v}$ and modifying the route of a vehicle. Under these scenarios, the vehicle might choose to proceed on its own, resulting in saving time. Therefore, the computation of $T_{d}$ empowers the vehicle to make well-informed choices regarding coalition formation by weighing the benefits against the associated costs.
- ***Overlapping Distance—***The overlapping route $O_{V↔C}^{D}$ pertains to a specific route of length $L_{r}$, which both the vehicle V and the coalition C can simultaneously travel in the same direction $\overset{\mathrm{dir}}{\to }$. To ensure safe and efficient travel within the coalition C, all vehicles must be synchronized and adhere to this shared route. Therefore, to calculate the $O_{V↔C}^{D}$, the V utilizes various sensor devices to gather and communicate essential data. This information includes speed V of the vehicle, position $P_{v}\left(u,v\right)$, and the planned traveling route $R_{v}$ with neighboring vehicles $N_{v}$ or with the coalition C if it is already formed. The traveling route $R_{v}$ of V is compared and synchronized with the route $R_{C}$ of the coalition C, and their similarities are used to determine the $L_{r}$ of the road segment where their routes overlap.
  In complex urban settings, the overlapping distance $O_{V↔C}^{D}$ may only span a short distance before vehicles diverge onto separate routes. However, in the controlled highway scenarios, the $O_{V↔C}^{D}$ encompass a substantial segment of the route, influenced by factors such as $T_{d}$, etc.
- ***Speed Difference—***The computation of the speed variation $\Delta V_{dif}$ between the vehicle and the coalition is calculated using Equation (31). This equation assesses the variance between the desired travel speed *V* for vehicle V on the road and the current *V* maintained by the vehicles in the coalition C.
  (31) $$\Delta V_{dif}=\left\{\begin{array}{cc} 1-\frac{\left|V_{des}-V_{C}\right|}{V_{des}} & \;\;\;\;V_{Min}\leq V_{C}\leq V_{Max}\\ 0 & \;Otherwise \end{array}\right.$$
  In Equation (31), $V_{des}$ denotes the speed that vehicle V aims to achieve; $V_{C}$ denotes the speed provided by the C to V; $V_{Min}$ and $V_{Max}$ are the minimum and maximum speed limits for V, respectively. It is important to note that, a lower value of $\Delta V_{dif}$ is considered more favorable for V. For instance, if the $V_{des}$ of V exceeds the offered speed $V_{C}$ (e.g., $V_{des}$ = 45 km/h and $V_{C}$ = 35 km/h), V might not find it appealing to join the coalition due to the lower offered speed. Conversely, if $V_{des}$ is lower than $V_{C}$ (e.g., $V_{des}$ = 30 km/h and $V_{C}$ = 45 km/h), it may become more attractive for V to be part of the coalition, as it allows for quicker arrival at the destination. However, it is also worth mentioning that while speed is a key factor, other considerations, such as fuel efficiency and maintaining safe and optimal driving conditions, should also be taken into account by V when deciding whether to join the coalition.

#### 4.3.2. Objective Function

The objective function plays a vital role in modeling and assessing the benefits attained by V during its journey within the coalition C. In this work, we formulate and characterize the objective function, denoted as Gain, by taking into account various incentives and benefits as presented in Equation (32). The aim is to model these incentive functions simply while capturing all the relevant parameters. The suggested Gain function is divided into five components: (i) $D_{ci}$: calculates the car insurance discount that a vehicle *V* can receive while driving within C; (ii) $D_{tf}$: computes the traffic fine discount when the V travels within C; (iii) $D_{toll}$: calculates the toll discount for the vehicles traveling within C; (iv) FC: calculates the fuel consumption of *V* when travels within C; and (v) TT: calculates the travel time that a vehicle will take to reach the destination while traveling within C. The weighting coefficients $\gamma_{1}$, $\gamma_{2}$, $\gamma_{3}$, $\gamma_{4}$, and $\gamma_{5}$ reflect the influence of each component, and their total add upto 1 as expressed by $\sum_{i=1}^{n}\left(\gamma_{i}\right)=1$.

(32) $$Gain=\gamma_{1}\times D_{ci}+\gamma_{2}\times D_{tf}+\gamma_{3}\times D_{toll}+\;\gamma_{4}\times F_{C}+\;\gamma_{5}\times \mathrm{TT}$$

Subsequently, we delve into a detailed discussion of each component.

- ***Car Insurance Discount—*** Car insurance discounts serve as a powerful incentive mechanism for vehicles, motivating them to travel in coalition. To encourage this behavior, authorities can call upon insurance companies to offer discounts to collaborating vehicles, provided they adhere to specific criteria, such as the minimum distance, accident history, and coalition size. We believe that this initiative can yield numerous benefits, benefiting both individual vehicles and the environment as a whole.
  We model this function by defining a base discount rate $D_{br}$ that is assigned to a vehicle V under the given criteria and conditions. Vehicles have to travel a minimum distance $D_{M}$ in kilometers in the coalition C to be eligible for the car insurance discount. This requirement ensures that platooning remains safe and effective. The next step in determining the $D_{ci}$ is to compute the additional distance $D_{A}$ traveled beyond the $D_{M}$ required distance, expressed as:
  (33) $$D_{A}=max\left(D_{T}-D_{M},0\right)$$
  In Equation (33) the function calculates the difference between the total distance $D_{T}$ traveled in the coalition C and the $D_{M}$ that should be traveled in the C to benefit from the discount. The additional discount for $D_{A}$ is then calculated based on the additional distance traveled and a discount rate per distance interval.
  (34) $$D_{a}=\left(\frac{D_{A}}{5}\right)\times D_{i}$$
  In Equation (34), the additional distance $D_{A}$ is divided by 5, representing intervals of 5 km, and then multiplied by the discount rate per interval $D_{i}$.The second criterion for computing $D_{ci}$ is to evaluate the accident history. In Equation (35), a 0.05 discount is applied if there have been no accidents while traveling in a coalition; otherwise, no additional discount is given. This discount can be represented as follows:
  (35) $$D_{na}=\left\{\begin{array}{cc} 0.05 & \mathrm{if}\;\mathrm{no}\;\mathrm{accident}\\ 0 & \mathrm{Otherwise} \end{array}\right.$$
  Conversely, if a vehicle is involved in an accident while traveling in a coalition, the accident penalty $A_{p}$ is applied, as expressed in Equation (36).
  (36) $$A_{p}=\left\{\begin{array}{cc} 0.05 & \mathrm{if}\;\mathrm{there}\;\mathrm{is}\;\mathrm{an}\;\mathrm{accident}\\ 0 & \mathrm{Otherwise} \end{array}\right.$$
  An additional discount, denoted as $D_{cs}$, is provided based on the third criterion. A discount of 0.02 is applied if the size of the coalition CS falls within the range $4<\mathrm{CS}\leq {\mathrm{CS}}_{max}$. If CS exceeds ${\mathrm{CS}}_{max}$, there is a gradual decrease in the discount, as expressed in Equation (37). This component incentivizes vehicles to maintain the coalition size CS within the optimal range of four to six vehicles.
  (37) $$D_{cs}=\left\{\begin{array}{cc} 0.02 & if\;4\;\leq \;\mathrm{CS}\;\leq {\mathrm{CS}}_{max}\\ (\mathrm{CS}-{\mathrm{CS}}_{max})\times 0.01 & if\;\mathrm{CS}>{\mathrm{CS}}_{max}\\ 0 & Otherwise \end{array}\right.$$
  Finally, the total car insurance discount $D_{ci}$ for collaborative vehicles is determined by combining all these factors in Equation (38).
  (38) $$D_{ci}=D_{br}+D_{a}-A_{p}+D_{na}+D_{cs}$$
- ***Traffic Fine Discount—*** Traffic fine discounts can offer tangible financial benefits to vehicles that choose to drive in coalitions, directly reducing the cost of driving and making convoy driving an economically attractive option.
  We introduce a traffic fine discount function, serving as an incentivizing mechanism implemented by transportation authorities to encourage vehicles to travel in coalitions. This function promotes convoy driving by offering varying financial incentives to drivers based on their adherence to the time of day, coalition size, and speed restrictions. By providing these discounts, transportation authorities seek to optimize traffic flow, reduce congestion, and enhance road safety throughout the day. Essentially, this represents a proactive strategy to address traffic-related issues, decrease fuel consumption, and improve overall traffic flow by rewarding collaborative driving behavior and fostering a more efficient and harmonious traffic environment. Subsequently, we discuss the formulation of the traffic fine discount function, denoted as $D_{tf}$.
  Let P be a binary variable that refers to specific periods during the day when traffic congestion is either at its highest (peak time) or lowest (off-peak time), then the base discount rate $D_{br}$ can be written as:
  (39) $$D_{br}=\left\{\begin{array}{cc} 0.25 & \mathrm{if}\;P=1\\ 0.20 & \mathrm{if}\;P=0 \end{array}\right.$$
  Equation (39) models the $D_{br}$, depending on whether the coalition is formed during peak or off-peak times. A higher $D_{br}$ is assigned to vehicles when they form coalitions during peak times, leading to improved traffic flow and reduced congestion. Furthermore, to maintain traffic stability, we consider the coalition size, CS, and introduce a gradual decrease in the discount as CS increases beyond the maximum coalition size, ${\mathrm{CS}}_{max}$. Equation (40) reduces the discount rate by 0.02 for each additional vehicle beyond ${\mathrm{CS}}_{max}$.
  (40) $$D_{cs}=\left(\mathrm{CS}-{\mathrm{CS}}_{max}\right)\times 0.02$$
  To adjust the discount based on average coalition speed $V_{a}$ compared to an acceptable speed range, we employ specific conditional statements. Given the constraints of the urban environment, we maintain the acceptable speed range for coalitions between 50 km/h and 55 km/h. Penalties are applied for speeds beyond this acceptable range, as formulated in Equation (41). Equation (42) combines all the factors to calculate the total $D_{tf}$.
  (41) $$D_{v}=\left\{\begin{array}{cc} 0 & \mathrm{if}\;50\leq V_{a}\leq 55\\ 0.03 & \mathrm{if}\;V_{a}<50\\ 0.05 & \mathrm{if}\;55<V_{a}\leq 65\\ 0.07 & \mathrm{if}\;65<V_{a}\leq 75\\ 0.09 & \mathrm{if}\;V_{a}>75 \end{array}\right.$$
  (42) $$D_{tf}=max\left(D_{br}-D_{cs}-D_{v}\;,\;0\right)$$
- ***Toll Discount—***To motivate vehicles to travel in coalitions and incentivize them with toll discounts, authorities can implement a toll discount policy that encourages and rewards collaborative behavior. However, the vehicles have to adhere to specific criteria, such as maintaining the coalition size CS and the average speed $V_{a}$.
  Let $D_{toll}$ be the total toll discount amount, $V_{a}$ be the average coalition speed and CS be the size of the coalition then the $D_{toll}$ can be formulated as:
  (43) $$D_{toll}=T+D_{t}$$
  In Equation (43), *T* represents the base toll amount, while $D_{t}$ represents the total discount rate. This rate is computed as a combination of the base discount ($D_{br}$) rate, the discount adjustment factor ($D_{af}$), and the speed discount ($D_{v}$).
  (44) $$D_{t}=D_{br}+D_{af}+D_{v}$$
  The $D_{br}$ is based on the coalition size, and the maximum discount is granted when CS falls within the desired acceptable range.
  (45) $$D_{br}=\left\{\begin{array}{cc} 0.20 & \mathrm{if}\;4\leq \mathrm{CS}\leq {\mathrm{CS}}_{\max}\\ 0.17 & \mathrm{if}\;\mathrm{CS}\geq {\mathrm{CS}}_{\max} \end{array}\right.$$
  The $D_{af}$ represents an adjustment to the discount rate based on coalition size exceeding ${\mathrm{CS}}_{max}$. It functions as a penalty for larger coalitions, reducing the discount rate by 0.02 for each additional vehicle beyond ${\mathrm{CS}}_{max}$, effectively penalizing larger coalitions.
  (46) $$D_{af}=\mathrm{CS}-{\mathrm{CS}}_{max}\times -0.02$$
  The $D_{v}$ represents the speed-based discount rate. A 0.02 discount is applied when the speed falls within the acceptable range; otherwise, penalties are imposed as the speed exceeds the acceptable range.
  (47) $$D_{v}=\left\{\begin{array}{cc} 0.02 & \mathrm{if}\;50\leq V_{a}\leq 55\\ -0.03 & \mathrm{if}\;55\leq V_{a}\leq 65\\ -0.06 & \mathrm{if}\;65\leq V_{a}\leq 75 \end{array}\right.$$
- ***Travel Time—*** Traveling within a coalition C offers substantial benefits over traveling individually, primarily in terms of significantly reducing the time required to arrive at *d*. This advantage arises from the tighter inter-vehicle distance within the coalition, which is less than the distance required for vehicles traveling independently along the identical route. By decreasing the spacing among vehicles, the coalition improves traffic flow, leading to reduced travel times. Consequently, vehicles reach their destination *d* faster compared to if they are traveling individually. The proposed function for estimating the travel time TT of a vehicle V as it travels within a coalition C is presented below:
  (48) $$\mathrm{TT}=\frac{\left(D+\left(\mathrm{CS}-1\right)\times T_{f}\right)}{V_{a}}\;+\left(N_{s}^{\;C}\times S_{t}^{\;C}\right)$$
  In Equation (48), $T_{f}$ denotes the inter-vehicle spacing between the followers of the coalition C; $V_{a}$ is the average speed of the vehicles in the C; $N_{s}^{C}$ is the count of stop(s) that the C may make on the way to *d*; and the $S_{t}^{C}$ signifies the duration of time that coalition C spends at each stop. The smaller the $T_{f}$, the faster C reaches its destination *d*.
- ***Fuel Consumption—*** One of the primary incentives to travel in C is to achieve significant fuel savings. Traveling in C is more fuel-efficient, especially on long journeys. This efficiency stems from a reduction in aerodynamic resistance, a significant contributor to fuel consumption when traveling at high speeds.
  As a vehicle V moves on its own, it disturbs the air in its vicinity, resulting in a pressure disparity extending from the front to the rear. This pressure differential gives rise to an aerodynamic drag force $F_{A}$, which acts to impede the vehicle’s onward movement. The magnitude of this drag force grows proportionally to the square of the vehicle’s velocity. Consequently, as the vehicle accelerates, the $F_{A}$ becomes increasingly pronounced. On the contrary, when vehicles move within the coalition C, the front vehicle pierces through the air, creating an area of reduced pressure in its wake. Successive vehicles in coalition C travel within this reduced pressure area, which diminishes their $F_{A}$. Consequently, vehicles traveling in a C require less fuel to sustain their speed V compared to when they are traveling individually.
  Many fuel consumption models have been explored in the literature for estimating fuel consumption, including the ARRB [28], VSP [29], and MEF [30]. In this work, we employ the VT-Micro vehicle-based model to compute the fuel consumption of V traveling within C based on instantaneous speed *V* and instantaneous acceleration *a*. The model is discussed in Equation (25) and the regression coefficients are presented in Appendix A Table A3. Furthermore, the readers are encouraged to look into the authors’ recent publications [31] for the physics-based model, where we simulate the aerodynamic drag component based on varying inter-vehicle spacing and its effect on fuel consumption, which is essential to calculate the fuel consumption of coalition.

#### 4.3.3. Cost Function

The cost function $J_{C}$ comprised of various factors is designed to capture the cost that a vehicle V has to incur in aiming to join the coalition. The proposed $J_{C}$ is presented in the below equation:

(49) $$J_{C}=\beta_{1}\times \psi_{cost}^{LC}+\beta_{2}\times \psi_{cost}^{CT}+\beta_{3}\times \psi_{cost}^{SS}$$

In Equation (49), the function is comprised of three main components such as the lane change cost $\psi_{cost}^{LC}$, catch-up cost $\psi_{cost}^{CT}$, and the speed synchronization cost $\psi_{cost}^{SS}$. The weighting coefficients $\beta_{1}$, $\beta_{2}$, and $\beta_{3}$ reflect the influence of each component, and their total adds up to 1 as expressed by $\sum_{i=1}^{n}\left(\beta_{i}\right)=1$.

Subsequently, we delve into a detailed discussion of each component of $J_{C}$:

- ***Lane Change Cost—*** The cost linked to lane changes, symbolized as $\psi_{cost}^{LC}$ for vehicle V, signifies the expense accrued when switching to another lane, referred to as $\hat{L}$ to become a part of the coalition C. This cost is computed by taking into account a range of factors, including the distance traveled to change lanes, the number of lane changes, and the potential collision risk.
  We design a piecewise function, defined in Equation (50), to calculate the $\psi_{cost}^{LC}$ cost. based on two parameters, *X* and $N_{lc}$.
  (50) $$\psi_{cost}^{LC}=\left\{\begin{array}{cc} 0 & \;\;N_{lc}=0\\ b\times X+c & \;\;N_{lc}=1\\ K\times X^{2} & \;\;N_{lc}>1\; \end{array}\right.$$
  In Equation (50), the variable *X* denotes the distance that V needs to cover in order to change lanes and merge with C; $N_{lc}$ represents the count of lane changes necessary for vehicle V; the coefficients *b* and *c* are utilized in the linear part of the cost function, reflecting the cost associated with lane changes over a certain distance and the symbol K stands for the coefficient in the quadratic part of the cost function, denoting the cost associated with lane changes over a squared distance. Incorporating the lane-changing cost into the comprehensive cost function enables vehicles to make more educated choices when determining whether to become part of coalition C taking into account the associated risks and expenses associated with changing lanes.
- ***Catch-up Cost—*** The vehicles are spatially scattered on the road network. The cost to catch up with the coalition denoted as $\psi_{cost}^{CT}$ represents the time that the vehicle V takes to join C, and it is intricately linked to the speed V and the longitudinal gap between the vehicle V and the target coalition C. The proximity between the two plays a pivotal role in determining the feasibility of the merging process. The $\psi_{cost}^{CT}$ is modelled as presented in Equations (51) and (52) below:
  (51) $$L_{V↔C}^{G}=\left|P_{v}\left(u,v\right)-P_{C}\left(u,v\right)\right|$$
  (52) $$\psi_{cost}^{CT}=\frac{L_{V↔C}^{G}}{V_{rel}}$$
  Firstly, in Equation (51) the longitudinal gap $L_{V↔C}^{G}$ is calculated by taking the difference between the position of V denoted as $P_{v}\left(u,v\right)$ and the C denoted as $P_{C}\left(u,v\right)$. Equation (52) calculates the $\psi_{cost}^{CT}$ by dividing the $L_{V↔C}^{G}$ to the relative speed $V_{rel}$ where the $V_{rel}=V_{cur}^{v}-V_{cur}^{C}$. A shorter distance to the target C leads to a lower cost of time, as it reduces the time required for V to position itself within the coalition’s formation. Conversely, a greater distance may result in increased time and fuel spent to synchronize the vehicle’s speed and trajectory with that of C, emphasizing the critical interplay between distance and the associated cost of time in the context of coalition formation.
- ***Speed Synchronization Cost—*** This represents the cost experienced by a vehicle V when becoming a part of the coalition C necessitating synchronization of its speed with C. Specifically, if the coalition C is moving at a faster speed *V* than the vehicle V attempting to join, the vehicle V might need to accelerate to match the speed of C in order to become part of the coalition. This adjustment results in elevated fuel consumption and heightened risk for vehicle V, consequently incurring a cost for the vehicle. Consequently, it is important to quantify the speed synchronization cost based on the speed V disparity between the vehicle and the coalition.
  (53) $$\psi_{cost}^{SS}=\Gamma \times \frac{\left|V_{cur}^{v}-V_{cur}^{C}\right|}{V_{cur}^{v}}$$
  In Equation (53), the $V_{cur}^{v}$ denotes the current speed of the V, $V_{cur}^{C}$ is the current speed of the coalition C. The value of $\psi_{cost}^{SS}$ is set to zero when vehicle V is already a part of coalition C. This cost element considers practical factors, such as the need for changes in speed to become a part of C, and its magnitude relies on several factors, including the speed disparity between the current speed of the coalition $V_{cur}^{C}$ and the current speed of the vehicle $V_{cur}^{v}$. Through experimentation, we observe that constant values within the range of 0.05 to 0.1 are suitable for the parameter Γ.

## 5. Evaluating the Collaborative Driving Game with Shapley Allocation Analysis

The output of a coalitional game is to distribute the payoffs among the players, ensuring that the benefits are perceived as equitable. In this research, we utilize Shapley allocation [32], a widely recognized solution concept, to fairly distribute the payoffs among the players of the coalition. Next, we delve into a detailed discussion of Shapley allocation.

Distributing the payoff evenly is insufficient, as it does not consider the different contributions of each member to the C. The Shapley value is a solution concept that ensures a fair distribution of the overall utility generated by a coalition C among its individual players in the set N. Computing the prospective payoff for all participants within N is crucial to inform their decision-making when joining the coalition. To compute the Shapley values requires examining all conceivable arrangements of vehicles within the coalition and analyzing the incremental contributions of each vehicle as they become members or depart from the coalition C. This procedure aids in determining the individual contributions that each player offers to the coalition C such as different discounts, fuel savings, minimized travel time, and other advantages.

The Shapley allocation ϕ(j) of an individual player *j* within a coalitional game G=〈N,ν〉, is computed as presented in Equation (54) [33].

(54) $$\phi \left(j\right)=\sum_{C\subseteq N\setminus \{j\}}\frac{|C|!(N-|C|-1)!}{N!}\left(v\left(C\cup \left\{i\right\}\right)-v\left(C\right)\right)$$

In Equation (54), the v(C) denotes the payoff obtained by the coalition; C represents a coalition that excludes player *j*; $C\cup \left\{j\right\}$ denotes the coalition created by incorporating player *j* into the C; the symbol ∑ signifies a summation performed over all possible coalitions C that do not include player *j*; $\left|C\right|$ denotes the count of players within C, and N denotes the overall count of participants in the game. Furthermore, to evaluate the game, we model the Shapley value with the symmetric axiom ensuring the fair distribution of payoff. The symmetric axiom states that two players, denoted as *i* and *j*, contributing equally to a coalition C in a game G, should obtain equal Shapley values.

In order to analyze the proposed collaborative driving game, we simulate a coalition consisting of six players, and the CAV penetration rate is set to 1. The parameter values employed for simulating the game are shown in Table 3. The experimental findings, as presented in Table 4, illustrate the Shapley values (ϕ) associated with all the vehicles when they operate within a coalition C. These findings clarify the Shapley value, representing the unique contributions of each vehicle to the collective value of the coalition. These contributions are evaluated in terms of various factors, including discounts, enhancements in fuel efficiency, and reductions in the coalition’s travel time. The outcomes underscore that vehicles with higher ϕ yield greater influence and make more substantial contributions to the outcomes of the coalition. Notably, the results highlight that Vehicle $V_{6}$ obtained the highest ϕ of 4.85 primarily attributed to factors such as its highest overlapping distance with the coalition and reduced joining coalition costs.

## 6. Numerical Experiments and Evaluation

In this section, we delve into the experimental configuration and discuss the numerical outcomes derived from a comprehensive series of experiments created to evaluate and confirm the efficacy of the suggested approach while also evaluating the benefits of traveling in coalition formation for both the societal and at the individual vehicle level.

### 6.1. Societal Benefits of Convoy Driving

The experiments are carried out to assess the impact of coalition-driven travel on traffic flow optimization and pollutant emissions. The parameters used to simulate the experiments are presented in Table 3.

#### 6.1.1. Traffic Flow

This section presents a series of experiments to investigate in detail how the coalition size (CS), coalition intensity (CI), CAV penetration rate $\left(P_{r}\right)$ and free-flow speed $\left(V_{f}\right)$ affect traffic flow, density, and speed within the fundamental diagram model. All the experiments are derived from the modeling approach detailed in Section 3.1.

**Figure 4:**
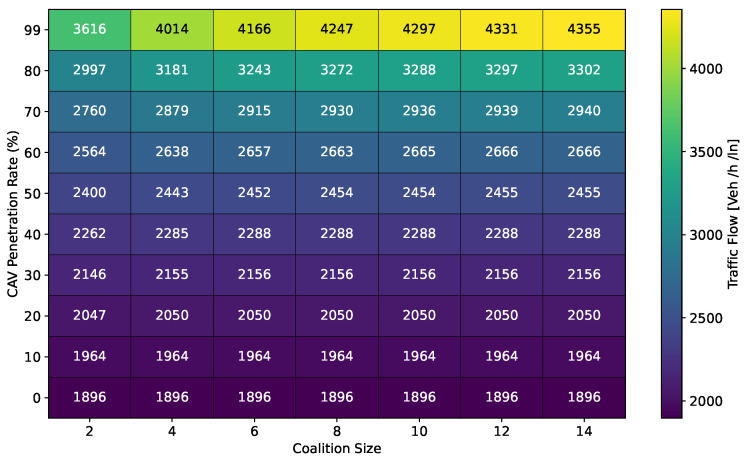
Impact of coalition Size and CAV penetration rate on traffic flow (CI=1.0).

**Figure 5:**
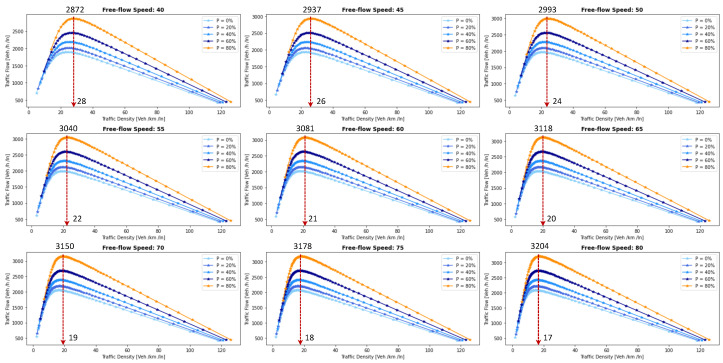
Impact of varying free-flow coalition speeds on traffic flow and density.

**Figure 6:**
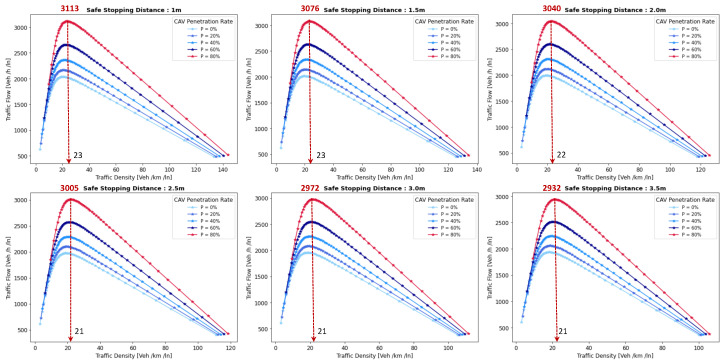
Impact of safe stopping distance on traffic flow and density.

**Figure 7:**
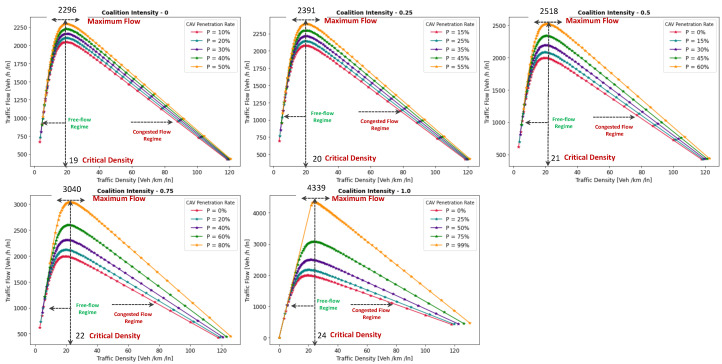
Traffic flow-density relationships for various coalition intensities and CAV penetration rates.

**Figure 8:**
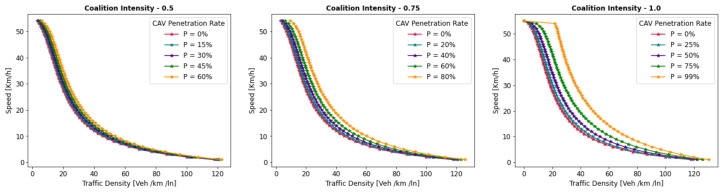
Impact of Coalition Intensity on Speed-Density Relationship.

**Figure 9:**
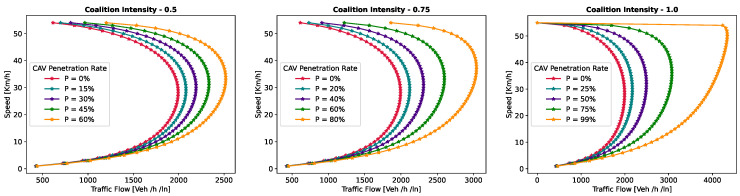
Speed-Flow relationship for various Coalition intensities and CAV penetration rates.

- ***Optimal Coalition Size—***In this experiment, we aim to find the optimal size of a coalition in an urban environment considering different crucial parameters. When the intensity of forming a CAV coalition is low, coalitions are less likely to reach their maximum size. Conversely, when coalition intensity is relatively high, most coalition can attain their maximum size. To investigate the impact of coalition size ranging from (2–14) vehicles on traffic flow considering varying $P_{r}$, we set the coalition intensity CI = 1.0 and incrementally increase CAV penetration from 0% to 99% with a step size of 10.
  The experimental results presented as a heatmap in Figure 4 reveal that the highest traffic flow measured in vehicles per hour per lane (veh/h/ln) is achieved when both coalition sizes and CAV $P_{r}$ are relatively high. Increasing coalition size exhibits a gradual improvement in traffic flow capacity. The larger the coalition size, the higher the traffic flow. The heatmap also illustrates that higher $P_{r}$ correlates with improved capacity, as indicated by the darker colors in the upper section of the map indicating that an increasing number of CAVs positively impacts the traffic flow. However, an intriguing observation is the diminishing returns associated with increasing coalition size beyond a certain threshold. While a larger coalition initially enhances traffic flow capacity, there comes a point where further increasing the coalition size beyond six has a marginal impact on traffic flow, and increasing the CAV penetration rate becomes imperative to increase traffic volume. Therefore, based on these findings, we conclude that the optimal coalition size, while considering the limitations of the urban environment and maintaining traffic flow stability, is 4–6, and we use this as the maximum coalition size in all the subsequent experiments.
- ***Free Flow Speed—*** Free-flow speed $V_{f}$ refers to the maximum speed at which vehicles can travel when there is no congestion or hindrance. In this experiment, we investigate the impact of varying free-flow speeds of coalition ranging from 40 km/h to 80 km/h with an increment of 5 km/h, on traffic flow and traffic density.
  The result presented in Figure 5 shows the optimal traffic flow and density for each $V_{f}$. It demonstrates a strong positive correlation between $V_{f}$ and traffic flow. As the $V_{f}$ increases, the capacity of the roadway to accommodate vehicles also rises. This relationship is intuitive, as higher speeds allow vehicles to cover more ground in a given time, resulting in an increased flow of vehicles. Conversely, the relationship between $V_{f}$ and traffic density is inversely proportional. As $V_{f}$ increases, traffic density measured in vehicle per kilometer per lane (veh/km/ln) decreases. Higher $V_{f}$ encourage vehicles to maintain greater spacing between each other, reducing the potential for traffic congestion.
  Higher $V_{f}$ can alleviate congestion, reduce travel times, and enhance overall road network efficiency; however, lower traffic densities contribute to improved road safety and reduced pollutant emissions. Therefore, we conclude that, in a complex urban scenario, a free-flow speed of 55 km/h represents an optimal balance. It maximizes traffic flow while maintaining safe and manageable traffic densities.
- ***Safe Stopping Distance—*** In this experiment, we investigate the impact of varying safe stopping distances $S_{min}$ from 1 m to 3.5 m with an increment of 0.5 m on both traffic flow and traffic density under various CAV $P_{r}$ and the CI=0.75. In Figure 6, the x-axis represents traffic density in (veh/km/ln), while the y-axis depicts traffic flow in (veh/h/ln) and the results are showcased for different CAV $P_{r}$ ranging from 0% to 80%.
  The results show that increasing the $S_{min}$ between the coalition members tends to decrease traffic flow. A larger $S_{min}$ means that vehicles need more inter-vehicle distance to stop safely, resulting in fewer vehicles passing a particular point on the road within a given time frame, leading to a reduction in traffic flow. As the $S_{min}$ increases, traffic density, representing the number of vehicles per unit length of the road, tends to decrease. This decrease in traffic density is a direct outcome of the increase in inter-vehicle distance, with more significant safety margins causing vehicles to spread out, thereby reducing congestion. The findings of this research show that selecting the optimal safe stopping distance should strike a balance between safety, traffic efficiency, road conditions, and the level of automation in the vehicles on the road. Therefore, we conclude that a safe stopping distance of 2 m is optimal, achieving a balance between safety and traffic efficiency.
- ***Impact of Coalition Intensity and CAV penetration rate on Traffic Flow-Density Relationship—*** To investigate how the CAV $P_{r}$ impacts the fundamental diagram of mixed traffic flow, the coalition intensity (CI) is set to CI = [0, 0.25, 0.5, 0.75, 1]. For each value of CI, the maximum penetration rate is computed using Equation (2). The experimental results, presented in Figure 7, illustrate the relationship between traffic flow and traffic density under various conditions of CI and $P_{r}$. Additionally, for each value of CI, the maximum flow and the critical density are annotated on the graph.
  The term free flow regime in the graphs refers to a traffic state where the coalitions move smoothly and efficiently at or near their desired speeds. In contrast, the congested flow regime represents a traffic state characterized by high traffic density and reduced vehicle speeds. It is evident from Figure 7 that as the coalition intensity ranging from 0 to 1 increases, traffic flow generally improves across all levels of CAV penetration. However, the extent of traffic capacity growth varies at different coalition intensities, with a more pronounced impact observed when coalition intensity is (CI = 0.75 or 1.0). Higher coalition intensity results in a more organized and efficient traffic flow, reducing congestion and increasing the number of vehicles passing through a lane per hour. Furthermore, irrespective of coalition intensity, an increase in CAV penetration rate typically leads to improved traffic flow. The results also show that the maximum traffic flow of 4339 (veh/h/ln) at CI = 1.0 $P_{r}$ = 99% increases the traffic flow by 88.81% compared to the maximum traffic flow of 2296 (veh/h/ln) at CI = 0 and $P_{r}$ = 50%. The findings of this experiment suggest that achieving high traffic flow rates and reducing congestion necessitates a combination of both high coalition intensity and a high CAV penetration rate.
- ***Speed-Density Relationship—*** In this experiment, we investigate the speed–density relationships for high levels of coalition intensity, CI = [0.5, 0.75, and 1.0], and various levels of CAV penetration rates, ranging from 0% to 99%. The results presented in Figure 8 indicate that at low traffic densities, traffic typically moves at or near the free flow speed, which is the speed at which vehicles travel without congestion. However, as traffic density increases, congestion begins to impact traffic speed, resulting in a decrease in speed. Higher CAV $P_{r}$ have a notable positive effect on traffic flow and speed, especially at moderate to high traffic densities. CAVs play a crucial role in mitigating congestion and maintaining relatively higher speeds compared to non-CAV scenarios. This impact of CAV penetration on traffic speed is more pronounced at higher coalition intensity levels where CAVs significantly influence traffic flow and higher speeds. Therefore, we conclude that CAV coalitions have the potential to enhance traffic conditions by reducing congestion and maintaining higher speeds, especially in scenarios with greater platoon intensity.
- ***Speed-Flow Relationship—*** In this experiment, we investigate the speed–flow relationship for different coalition intensities (0.5, 0.75, and 1.0) under varying levels of CAV penetration rates, shedding light on how traffic speed changes with the flow of traffic on a roadway. The result presented in Figure 9 shows that the presence of CAVs has a significantly positive impact on traffic flow. Higher CAV penetration rates lead to more favorable speed–flow curves. As CAVs become more prevalent, traffic flow remains efficient, and vehicles can maintain higher speeds even as traffic density increases. However, this effect is most pronounced at higher coalition intensities, where CAVs play a pivotal role in achieving near-optimal traffic conditions and significant improvements in traffic flow and speed stability.

#### 6.1.2. Pollutant Emission

The CACC model is utilized to simulate a coalition of varying sizes to capture the trajectories of CAVs. As discussed in Section 3.2, the VT-Micro model computes vehicle transportation pollutant emissions and fuel consumption using trajectory data, which includes the vehicle’s position, instantaneous speed, and instantaneous acceleration at each simulation time step. To acquire the necessary data, a simulation is run for a duration of 1200 s, with a simulation time-step of 1 s.

**Figure 10:**
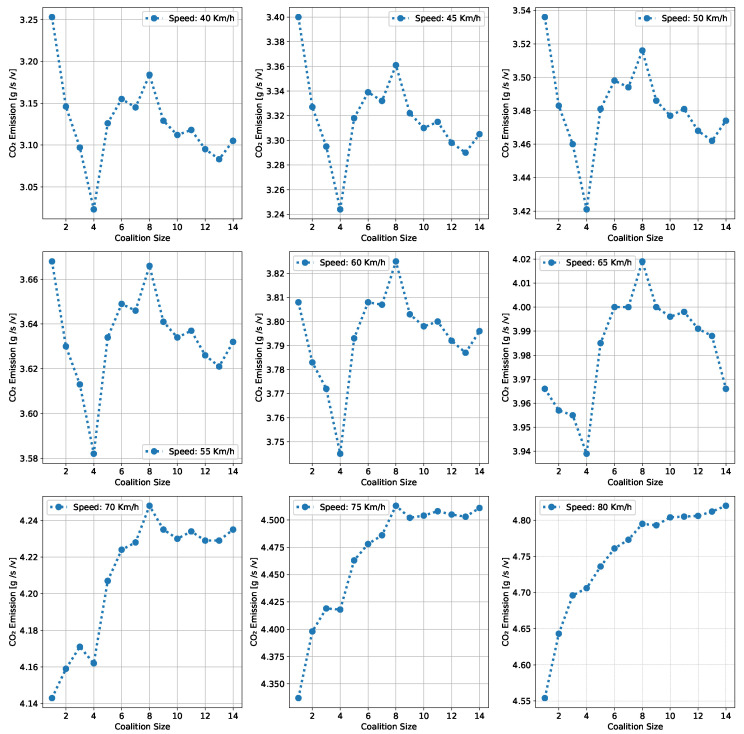
Impact of traveling in coalition on CO_2_ Emission at Various Speeds.

**Figure 11:**
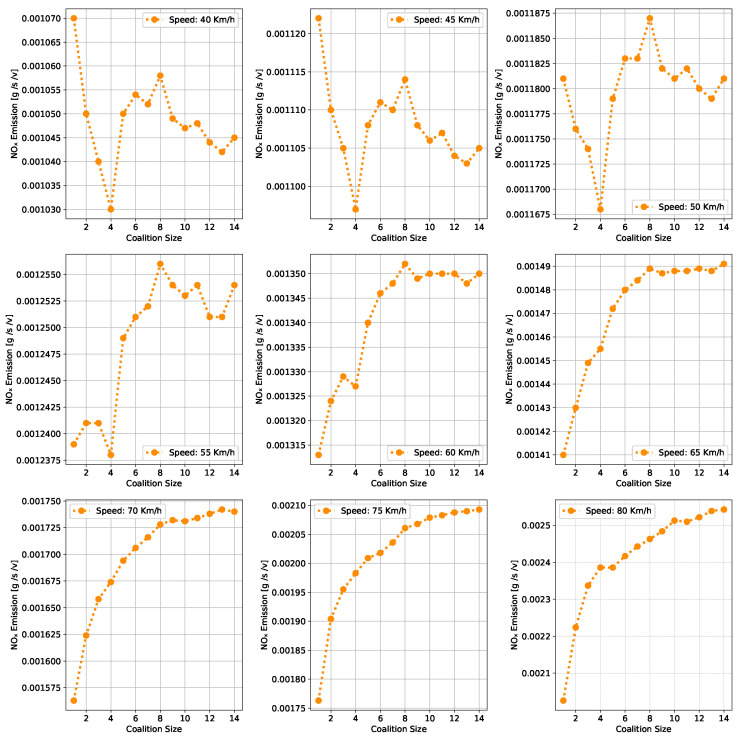
Impact of traveling in coalition on NO_x_ Emission at Various Speeds.

- ***Carbon Dioxide Emission—*** To evaluate the impact of coalition-driven travel on CO_2_ emission, the penetration rate of CAV is set to $P_{r}=1$. The instantaneous speed and acceleration at each time step are derived based on the simulation results. The experimental findings presented in Figure 10, depict that there is a clear correlation between speed and CO_2_ emissions. Lower speeds exhibit notably lower emissions, while higher speeds are associated with slightly increased emissions.
  At lower speeds, ranging from 40 to 55 km/h, the results show a noticeable decline in CO_2_ as the coalition size increases. This suggests that when vehicles travel at lower speeds and maintain a shorter following distance, reduced air resistance from drafting behind the lead vehicle improves fuel efficiency, resulting in lower CO_2_ emissions. Although the emissions exhibit slight fluctuations, it shows that forming larger coalitions at lower speeds consistently reduces CO_2_ emissions per vehicle. Furthermore, the reduction in CO_2_ is more significant when transitioning from a small coalition to a moderate-sized one, with the additional reduction becoming less pronounced as the coalition size further increases.
  In contrast, at higher speeds between 60 and 80 km/h, as the coalition size increases, CO_2_ tends to stabilize and show a mild increase. This phenomenon can be attributed to the diminished aerodynamic advantages of drafting at increased velocities. At these speeds, the advantages of reduced air resistance are outweighed by other factors such as increased engine load and fuel consumption. Consequently, CO_2_ per unit of time remains relatively constant and slightly rises with coalition size.
  The findings highlight the complex interplay between speed, coalition size, and emissions and demonstrate that convoy driving at both lower and higher speeds can lead to substantial reductions in CO_2_; however, the rate of reduction diminishes as the coalition size increases, suggesting that there is an optimal coalition size that maximizes the advantages of collaborative driving in terms of CO_2_ reduction. These results can assist policymakers in setting speed limits and promoting coalition-based transportation to reduce CO_2_ emissions.
- ***Nitrogen Oxide Emission—*** To assess how traveling in coalition affects the NO_x_ emission, the penetration rate of CAVs is set to $P_{r}=1$. The instantaneous speed and acceleration at each time step are collected using the CACC model. Observing the results presented in Figure 11 shows several trends emerge. Firstly, there is a noticeable general increase in NO_x_ as coalition size grows, regardless of speed. This suggests that as more vehicles join the coalition, the overall emissions tend to rise. However, the rate of increase varies with speed, with higher speeds typically resulting in steeper NO_x_ increases with coalition size.
  At lower speeds, from 40 to 50 km/h, a relatively consistent pattern of NO_x_ is observed. Emissions consistently remain low, hovering around 0.00103 to 0.00118 g/s/v across various coalition sizes. This suggests that forming coalitions at lower speeds has a minimal impact on NO_x_ as the differences between coalition sizes are marginal.
  Conversely, at higher speeds ranging from 55 to 80 km/h, the NO_x_ exhibits a more noticeable upward trend as the coalition size increases. Emissions gradually increase from approximately 0.00122 g/s/v at a coalition size of 1 to around 0.0024 g/s/v at a coalition size of 14. This indicates that coalition at higher speeds leads to higher NO_x_ likely due to increased engine load and the need for more frequent acceleration, which can occur in larger coalitions.
  The findings highlight that there is a certain balance to be struck between speed and coalition size for minimizing NO_x_ emissions as the relationship between these variables is not consistent across all speed ranges. Such insights are crucial for policymakers to make informed decisions regarding the implementation of platooning strategies aimed at achieving both efficiency and environmental sustainability in transportation systems.

### 6.2. Benefits of Coalition-Driven at Individual Vehicle Level

This section presents the numerical outcomes derived from an extensive series of experiments performed to assess and validate the influence of coalition formation on the advantages for instance decreased fuel consumption and travel time, and other discounts achieved at the individual vehicle level. These experiments are conducted by simulating a scenario involving a three-lane segment of an urban roadway, with the environmental complexity level configured at 0.99, and the CAV penetration rate set at 1. In these experiments, each vehicle operates independently at a speed of 45 km/h, aiming for a desired speed of 50 km/h. Building upon insights gained from the preceding Section 6.1.1’s findings, we employ an optimal coalition size of six vehicles, a coalition speed of 55km/h with a safe stopping distance of 2 m to examine the benefits of coalition-driven travel. The parameters used to simulate the experiments are presented in Table 3.

In the subsequent subsections, we discuss each coalition-driven benefit in detail:

#### 6.2.1. Aggregated Utility of Traveling in Coalition

In this experiment, we assess the validity of the proposed utility function for CAVs as outlined in Section 4.3. A coalition consisting of six vehicles is formed in the peak time hours with an accident-free scenario to analyze the aggregated impact of various factors on the utility of the function.

**Figure 12:**
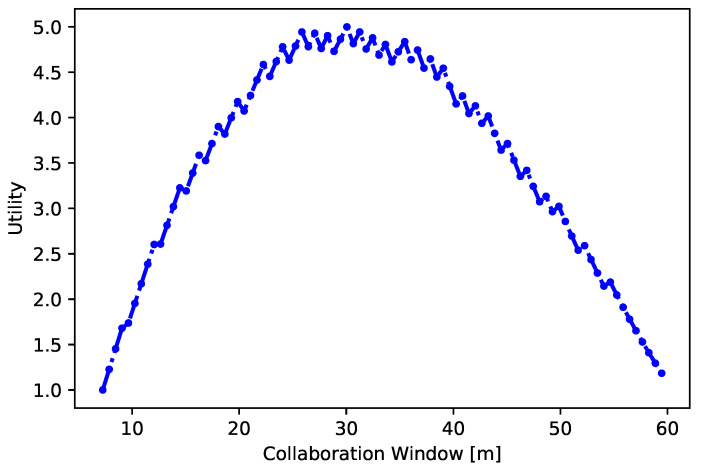
Relationship between the collaboration window and the aggregated utility of vehicle traveling in a coalition.

The result illustrated in Figure 12, depicts the relationship between collaboration window $C_{W}$ and the utility U that a vehicle V derives from its collaboration in the coalition. The x-axis illustrates the collaboration window which represents the time duration for which vehicles collaborate to achieve common objectives such as discounts and fuel consumption. Conversely, the y-axis signifies the utility value linked to $C_{W}$. The utility U quantifies the aggregated advantages that vehicles gain from collaborating in a coalition. It is computed based on factors including the collaboration window, objective function, and cost function (as discussed in Section 4.3). An elevated utility value indicates that the involved vehicle perceives a greater benefit. We normalize the utility U, scaling it within the range of $\left[1,5\right]$.

The results indicate that as the collaboration window increases, the utility also tends to increase. This suggests that a longer duration spent in a coalition generally leads to higher overall benefits for the vehicle. These benefits could include reduced travel time, discounts on traffic fines, tolls, and insurance, and lower fuel consumption. Additionally, the graph reveals a noticeable upward trend that appears to be relatively linear. This suggests a positive correlation between time spent in the coalition and utility, implying that collaborative driving tends to become more advantageous as the duration of collaboration increases. The oscillations in the result also demonstrate the variation in the U values for different collaboration window values. This suggests that utility values are influenced by factors other than solely $C_{W}$. These factors may be related to the speed of the C, overlapping distances, inter-vehicle spacing, coalition size, or other variables. It is important to note that the utility slowly begins to decline after 30 min of traveling in a coalition. This suggests that there is an optimal duration of collaboration in a coalition in the urban scenario. Beyond this optimal point, spending more time in the coalition may result in diminishing returns or even decreased utility for the vehicles.

#### 6.2.2. Discount on Car Insurance

**Figure 13:**
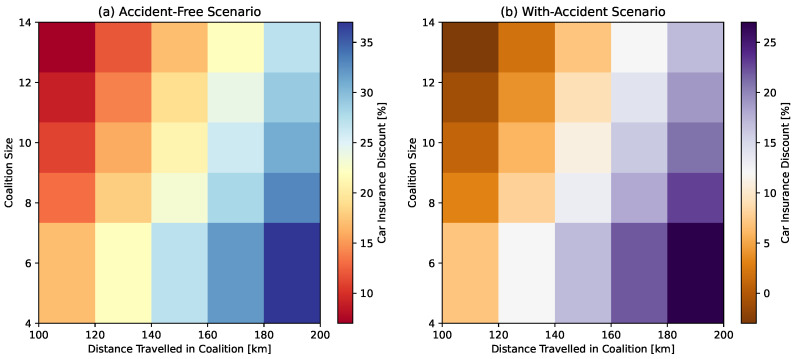
Car insurance discounts for accident-free and with-accident scenarios.

In this experiment, we investigate car insurance discounts for vehicles traveling in coalitions based on various factors, including coalition distance, coalition size, and accident history. The parameter values used in this experiment are presented in Table 3. Figure 13a visualizes car insurance discounts for collaborating vehicles under accident-free and with-accident scenarios. The x-axis represents the distance traveled within the coalition, ranging from the minimum required to the maximum value. The y-axis indicates coalition size, ranging from 4 to 14 vehicles.

In the accident-free scenario, it is observed that car insurance discounts generally increase as the distance traveled in the coalition increases. This suggests that vehicles covering longer distances in coalitions tend to receive higher discounts. For the optimal range of coalition sizes from four to six vehicles, insurance discounts increase from 17% to 35% as the total distance traveled increases from 100 km to 200 km. Smaller coalitions receive higher discounts, indicated by darker shades, incentivizing the maintenance of moderately sized convoys. Conversely, for larger convoy sizes (eight vehicles or more), discounts decrease as coalition size increases, regardless of the total distance traveled.

In Figure 13b, car insurance discounts for the with-accident scenario are visualized. In contrast to the accident-free scenario, discounts in this scenario are generally lower due to the occurrence of accidents, as indicated by lighter shades on the color scale. The reduction in discounts due to accidents is noticeable across all coalition sizes and distances. Similar to the accident-free heatmap, larger coalition sizes (eight vehicles or more) result in lower discounts, with the reduction due to accidents further impacting the discounts.

In conclusion, the results demonstrate that accident-free convoy driving is rewarded with higher discounts by the authorities, whereas accidents result in reduced incentives. Vehicles that commit to longer distances within the optimal coalitions can expect greater insurance discounts, assuming safety is maintained.

#### 6.2.3. Discount on Traffic Fine

In this experiment, we investigate the traffic fine discount offered to vehicles engaged in collaborative driving, taking into account different combinations of coalition size, coalition speed, and time of the day (peak time and peak-off times).

**Figure 14:**
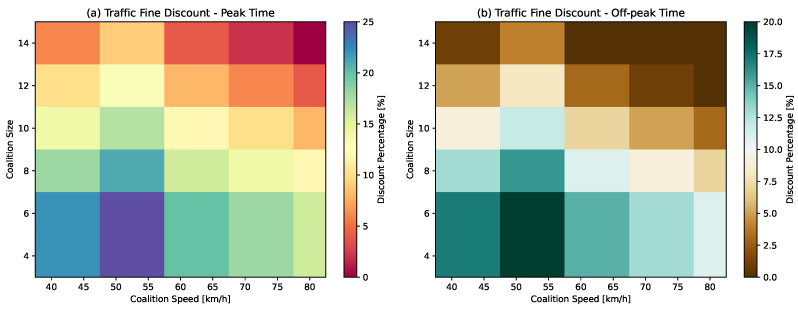
Traffic fine discounts for peak and off-peak times.

The heatmap presented in Figure 14 uses color-coded values to represent different discount percentages. The results show that in the peak-time scenario, Figure 14a, the smaller coalitions tend to receive higher discounts compared to larger ones. This trend is especially evident for coalition sizes up to six vehicles, after which the discount percentage remains relatively stable. This suggests that during peak hours, authorities are more inclined to offer larger discounts to encourage the smaller coalition formations aiming to uphold the stability of the urban setting. Discount percentages gradually decrease as coalition size exceeds six vehicles. The decrease is more noticeable for larger coalitions, indicating diminishing returns in terms of discounts as coalition size increases during peak hours. Coalition traveling within or near the acceptable speed range (50 to 55 km/h) tend to receive higher discounts. This is indicated by the regions in the heatmap where the discount percentage is relatively high and stable. Coalitions traveling below 50 km/h or above 60 km/h receive penalties, resulting in lower discounts. The results show that authorities encourage manageable-sized coalitions that adhere to speed limits to optimize traffic flow and emissions.

Similar to the peak time scenario, smaller coalitions with 4–6 vehicles typically receive higher traffic fine discounts during off-peak hours as shown in Figure 14b. However, the initial discount rate during off-peak hours is slightly lower, (approximately 20%) compared to peak hours. As coalition size increases beyond six vehicles, the discount percentage decreases, indicating diminishing returns. However, the penalties for speeds below the lower range and above the upper range may differ slightly from the peak time scenario.

Overall, the results provide insights into the incentive structure for collaborative driving during different times of the day. During peak hours, the focus is on encouraging smaller platoons with controlled speeds, while during off-peak times, incentives are still present but are less substantial.

#### 6.2.4. Discount on Toll

**Figure 15:**
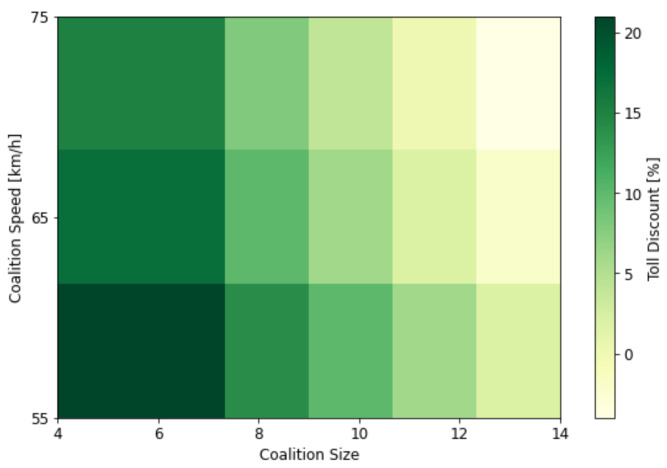
Toll discount for coalition-driven travel.

In this experiment, we investigate the toll discount based on the key variables of coalition sizes ranging from 4 to 14 and speeds (55, 65, 75 km/h). The result presented in Figure 15 employs a color spectrum ranging from green to yellow to convey valuable insights into toll policy dynamics. The findings indicate that smaller coalitions of 4–6 vehicles receive higher discounts. However, larger coalitions, those composed of seven or more vehicles, do not inherently receive higher discounts; instead, they are assigned a slightly lower base discount rate.

Furthermore, the results illustrate how coalition speed influences the toll discount landscape. Coalition traveling within an acceptable speed range of 50–55 km/h receives an additional discount, promoting the adoption of moderate speeds conducive to safety and fuel efficiency. However, exceeding speed limits above 55 km/h and 65 km/h results in penalties. These penalties serve as a proactive measure aimed at discouraging high-speed travel, which can be associated with safety risks and increased environmental impact.

In conclusion, the results underscore the nuanced interplay of coalition size and speed in shaping toll discounts and penalties. Policymakers can utilize these insights to craft more finely tuned toll policies that balance considerations of safety, fuel efficiency, and traffic management. Nevertheless, it is essential to note that the specific discount rates and penalty thresholds should be meticulously defined based on policy objectives.

#### 6.2.5. Time-Saving Benefits of Coalition Travel

In this experiment, we simulate a 60 km distance to investigate the impact of traveling within a coalition on travel time. We varied the coalition’s average speed from 40 km/h to 80 km/h in 5 km/h increments to cover a broad spectrum of scenarios and assess the performance at various speeds.

**Figure 16:**
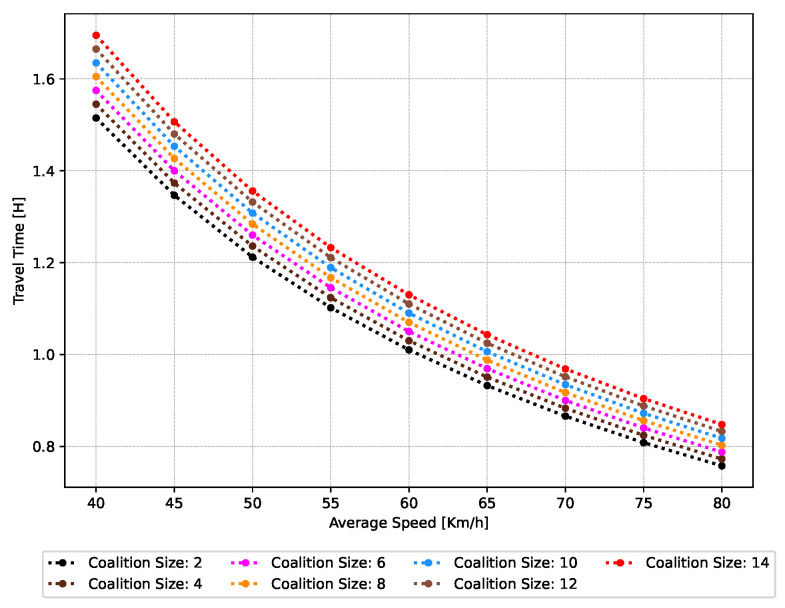
Effect of coalition-driven travel of varying sizes on travel duration.

The results depicted in Figure 16 illustrate an inverse correlation between the coalition’s average speed and travel time. With an increase in average speed, travel time diminishes. Notably, traveling within a coalition provides a significant advantage over traveling alone, particularly at higher speeds. Moreover, the results underscore the impact of coalition size on travel duration. For smaller coalitions consisting of two or four vehicles, the reduction in travel time is relatively gradual as speed increases, suggesting that at smaller group sizes, the impact of speed on travel time is less pronounced. In contrast, as the coalition size expands to include 6, 8, 10, and 12 vehicles, the reduction in travel time becomes more substantial at higher speeds. This indicates that larger coalitions benefit more from increased speeds in terms of travel time efficiency.

Additionally, the results reveal that the advantages of traveling within a coalition decrease significantly once the coalition size surpasses a certain threshold. This finding suggests a trade-off between the size of the coalition and the reduction in travel time underscoring the importance of carefully selecting the ideal coalition size to maximize the advantages of coalition-driven travel.

#### 6.2.6. Fuel Consumption Benefits in Coalition Travel

**Figure 17:**
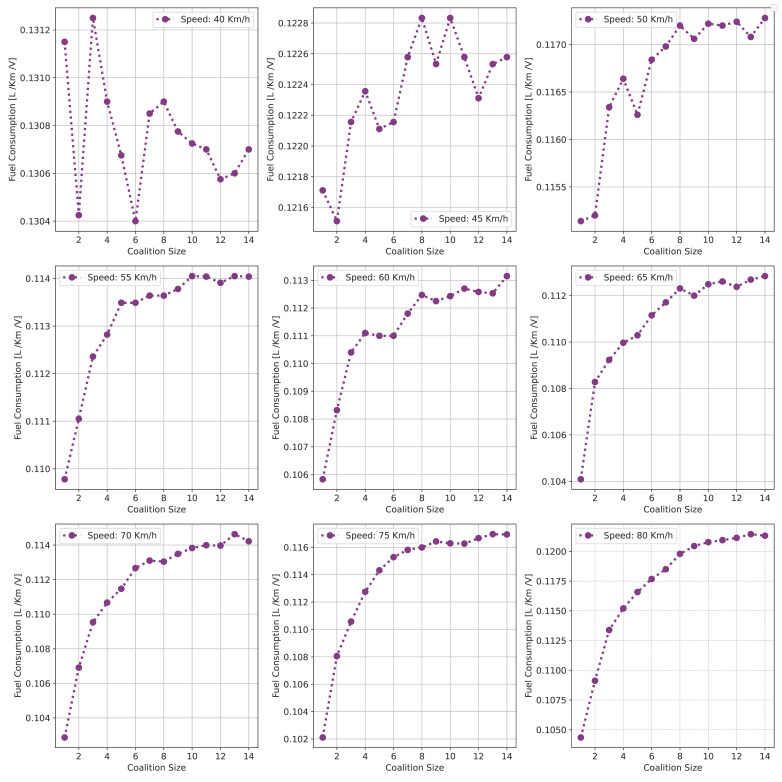
Impact of coalition size on fuel consumption at different speeds.

There are many studies [34,35] that investigate the impact of fuel consumption on CAVs; however, they did not consider the impact of coalition size on the fuel consumption of a vehicle. Therefore, in this study, we investigate the impact of coalition formation of different sizes (2, 3, 4,…, n) on average fuel consumption per vehicle, the instantaneous speed and acceleration are used, and the penetration rate of CAVs is set to $P_{r}=1$. The results presented in Figure 17 show that there is a direct correlation between speed and fuel consumption captured in liters per kilometer per vehicle (L/Km/V). As the coalition’s speed increases, fuel consumption (liter/km) rises proportionally. This finding aligns with the expectations, as higher speeds generally demand more energy, resulting in increased fuel consumption. Notably, the fuel consumption is at its lowest at 40 km/h and reaches its peak at 80 km/h.

At lower speeds, between 40 and 45 km/h, the results show a clear trend: as the coalition size increases, fuel consumption per vehicle tends to decrease. The reduction in fuel consumption is due to the significant impact of reduced aerodynamic drag that platooning provides. With vehicles closely following each other, the leading vehicle creates a smoother airflow for the trailing vehicles, leading to improved fuel efficiency. Therefore, for scenarios where vehicles are traveling at lower speeds, forming larger coalitions can result in notable fuel savings and increased overall efficiency.

However, at higher speeds, ranging from 50 to 80 km/h, the relationship between coalition size and fuel consumption is less pronounced. At higher speeds, the fuel consumption begins to rise slightly with extremely large coalition sizes. This shows that there are diminishing returns when adding more vehicles to a coalition. We conclude that identifying the optimal platoon size is crucial for practical implementation, balancing fuel savings with considerations of traffic flow and safety. While platooning can still yield some fuel savings at higher speeds, the differences between various platoon sizes are less pronounced compared to lower speeds.

## 7. Conclusions

In this research, we worked on collaborative convoy driving, aiming to propose a mutually beneficial solution for the authorities seeking sustainable transportation systems and for individual vehicle owners desiring explicit tangible benefits and incentives to participate in convoys. The main contribution of this research lies on two levels: modeling the societal benefits, such as optimized traffic flow and pollutant emissions, and the incentives at the individual vehicle level. We developed a fundamental diagram of mixed traffic flow, taking various factors such as CAV penetration rates, coalition intensity, coalition sizes, and free-flow speed into account to explore their relationships and their impact on traffic flow. First, the spatial arrangement of vehicles in mixed traffic flow, comprising HDVs and CAVs, is analyzed, and the results show that there are five types of car-following modes. Secondly, the coalition intensity at different levels is modeled, playing a crucial role in determining the varying degrees of clustering strength of CAVs. After that, we formulated the probability distributions of the car-following modes, considering both the coalition intensity and the CAV penetration rates. Then, a general framework for the fundamental diagram of mixed traffic flow is modeled. Pollutant emissions such as CO_2_ and NO_x_ are also modeled using the VT-Micro model to investigate the impact of convoys of varying sizes on emission reduction. The findings and the recommended policies for the authorities drawn from the numerical experiments are discussed below:

- When the intensity of forming a coalition is low, coalitions are less likely to reach their maximum size, and vice versa. The optimal coalition size is found to be 4–6 vehicles, which maintains the stability and safety of the urban environment. However, the coalition size has little effect on the flow when it exceeds 6.
- A higher free-flow speed has a positive impact on the maximum traffic flow in mixed-traffic settings. As free-flow speed gradually rises, the maximum traffic volume for mixed traffic also gradually increases, although the ideal traffic density decreases over time. The recommended optimal free-flow coalition speed is 55 km/h.
- The maximum traffic flow of mixed traffic is adversely affected by the safe stopping distance. As the minimum safe distance gradually increases, both the maximum traffic volume and the ideal traffic density for mixed traffic gradually decrease. The experiments show that 2 m is recommended to achieve a balance between safety and traffic efficiency.
- As the coalition intensity increases, traffic flow generally improves across all levels of CAV penetration. However, the extent of traffic capacity growth varies at different platoon intensities, with a more pronounced impact observed when coalition intensity is CI = 0.75 and 1.0.
- At low penetration rates, the intensity of connected and autonomous vehicle coalitions has a minor influence on the attributes of mixed traffic flow.
- The levels of CO_2_, NO_x_, and fuel consumption increase with higher speeds and larger coalition sizes.

Furthermore, we modeled the convoy driving problem using the coalition game framework and proposed a novel utility function encompassing incentives like car insurance discounts, traffic fine reductions, toll discounts, reduced travel time, and fuel consumption to encourage vehicles to participate in convoys. The proposed game is then analyzed using the Shapley allocation to fairly divide the payoff between the players of the coalition. The experiments showed that as the collaboration window expands, there is also a tendency for the utility to increase. This suggests that a longer duration spent in a coalition generally leads to higher aggregated benefits for the vehicles. Furthermore, it is evident from the results that the authorities are more inclined and rewarded when the coalitions adhere to the recommended optimal coalition size, speeds, and safe distance, and are formed during peak hours and travel accident-free. One of the limitations of the proposed study is that we did not consider modeling how different stakeholders, such as road traffic authorities, car insurance companies, etc., would engage and integrate to compute and provide incentives to the vehicles participating in the convoy driving. 

## Figures and Tables

**Table 1:** Comparative summary of the related work.

Ref.	Coalition Intensity	Coalition Size	Penetration Rate	Societal Benefits						Policy Formulation	Individual Benefits					
				Relationships & Characteristics of Fundamental Diagram				Pollutant Emission								
				Volume–Density	Speed–Density	Speed–Volume	Impact of Free Flow Speed	CO_2_	NO_x_		Car Insurance Discount	Traffic Fine Discount	Toll Discount	Fuel Consumption	Travel Time	Other
[6]	✓	✓	✓	✓												
[7]	✓	✓	✓	✓	✓	✓										
[8]		✓	✓					✓	✓					✓		
[9]	✓		✓	✓	✓	✓	✓									
[10]		✓	✓					✓	✓							
[11]	✓	✓	✓	✓												
[12]																✓
[13]														✓		✓
[14]																✓
[15]																✓
[19]	✓	✓	✓													
[20]		✓	✓													
[21]			✓	✓	✓											
[22]	✓		✓	✓												
This paper	✓	✓	✓	✓	✓	✓	✓	✓	✓	✓	✓	✓	✓	✓	✓	

**Table 2:** Headway spacing of mixed traffic in an equilibrium state.

Headway	Description
$H_{HDV}$ = $\frac{S_{min}+V_{e}\times T_{HDV}}{\sqrt{1-{\left(\frac{V_{e}}{V_{f}}\right)}^{4}+L_{v}}}$	Spacing between human driven vehicles.
$H_{a}=L_{v}+S_{min}+V_{e}\times T_{a}$	Spacing between CAVs traveling alone.
$H_{L↔HDV}=L_{v}+S_{min}+V_{e}\times T_{L↔HDV}$	Spacing between the CAV leader following an HDV.
$H_{L↔{\mathrm{CS}}_{max}}=L_{v}+S_{min}+V_{e}\times T_{L↔{\mathrm{CS}}_{max}}$	Spacing between the CAV leader following a CAV coalition.
$H_{m}=L_{v}+S_{min}+V_{e}\times T_{m}$	Spacing between coalition followers.

**Table 3:** Table of parameters and their values.

Parameter	Unit	Value	Description
$V_{cur}^{v}$	Km/h	45	Current speed of the vehicle
$V_{des}$	Km/h	50	Desired speed of the vehicle
$V_{cur}^{C}$	Km/h	55	Current speed of the coalition
V	Km/h	40–80	Coalition speed
$\lambda_{1}$, $\lambda_{2}$	-	0.35, 0.65	Weighing coefficients of $C_{E}$
$L_{v}$	m	5	Length of the vehicle
CS		6	Size of the coalition
$S_{min}$	m	2	Safe stopping distance
$T_{HDV}$	s	1.5	Spacing between HDVs
$T_{a}$	s	1.1	Spacing between CAVs traveling alone
$T_{L↔HDV}$	s	1.1	Spacing between leader of coalition and preceding HDV
$T_{L↔CS_{max}}$	s	1.0	Spacing between leader following a maximum-coalition
$T_{m}$	s	0.6	Spacing between the coalition followers
$C_{E}$		0.9	Complexity of the environment
Γ		0.1	Coordination parameter
ρ		0.6	$C_{W}$ coefficient
N		6	Number of vehicles in the coalition
$V_{f}$	Km/h	55	Free flow speed
$P_{r}$	%	0–100	CAV Penetration rate
CI		0–1	CAV Coalition Intensity
CS		2–14	Coalition size range
${\mathrm{CS}}_{max}$		6	Maximum coalition size
$D_{M}$	Km	100	Minimum distance
$V_{a}$	Km/h	55, 65, 75	Coalition Average Speed

**Table 4:** Shapley values of connected autonomous vehicles traveling in coalition.

$\phi_{\left(V1\right)}$	$\phi_{\left(V2\right)}$	$\phi_{\left(V3\right)}$	$\phi_{\left(V4\right)}$	$\phi_{\left(V5\right)}$	$\phi_{\left(V6\right)}$
3.0	3.5	3.48	4.0	4.3	4.85

## Data Availability

The parameters used in the simulation, along with their respective values, are provided in Table 3, offering a comprehensive reference for the experimental setup.

## Abbreviations

**Notation**	**Description**
V	Connected and autonomous vehicle
C	Coalition of CAVs
a(t)	Instantaneous acceleration of the vehicle
$V_{f}$	Free-flow speed of the vehicles
$V_{e}$	Equilibrium speed
$V_{a}$	Average speed of vehicles in C
$V_{n}\left(t\right)$	Speed of vehicle *n* at time *t*
$T_{m}$	Inter-vehicle spacing in the coalition
$P_{r}$	Penetration rate of CAVs
$P_{r}^{max}$	Penetration rate of CAVs
CI	Intensity of forming a coalition
$\hat{H}$	Average headway among vehicles in mixed traffic flow
K	Traffic Density
CS	Coalition size
$CS_{max}$	Maximum coalition size
Q	Traffic volume
$P_{e}$	Pollutant emission
$CO_{2}$	Carbon dioxide
$NO_{x}$	Nitrogen Oxides
$N_{v}$	Neighbouring Vehicles
$C_{W}$	Collaboration Window
$C_{E}$	Complexity of the environment
$O_{V↔C}^{D}$	Overlapping distance between the CAV and the coalition
$J_{C}$	Cost of joining a coalition
$S_{ec}$	Complexity of static elements in the environment
$D_{ec}$	Complexity of static elements in the environment
$L_{r}$	Length of the vehicle
$L_{r}$	Length of the route
$V_{des}$	Desired speed of CAV
$S_{V↔C}$	Longitudinal gap between the CAV and the coalition
$V_{rel}$	Relative speed
$V_{cur}^{C}$	Current speed of the coalition
$V_{cur}^{v}$	Current speed of the vehicle
ϕ(i)	Shapley value of player *i*

## Appendix A

**Table A1:** CO${}_{2}$ Coefficients for Positive and Negative Acceleration.

$R_{\mathrm{ij}}^{e}$	For a(t)≥0				$S_{\mathrm{ij}}^{e}$	For a(t)<0			
	j=0	j=1	j=2	j=3		j=0	j=1	j=2	j=3
i=0	6.916	0.217	2.35 $\times \;10^{-4}$	−3.64 $\times \;10^{-4}$		6.915	−0.032	−9.17 $\times \;10^{-3}$	−2.89 $\times \;10^{-4}$
i=1	0.02754	9.68 $\times \;10^{-3}$	−1.75 $\times \;10^{-3}$	8.35 $\times \;10^{-5}$		0.0284	8.53 $\times \;10^{-3}$	1.15 $\times \;10^{-3}$	−3.06 $\times \;10^{-6}$
i=2	−2.07 $\times \;10^{-4}$	−1.01 $\times \;10^{-4}$	1.97 $\times \;10^{-5}$	−1.02 $\times \;10^{-6}$		−2.27 $\times \;10^{-4}$	−6.59 $\times \;10^{-5}$	−1.29 $\times \;10^{-5}$	−2.68 $\times \;10^{-7}$
i=3	9.80 $\times \;10^{-7}$	3.66 $\times \;10^{-7}$	−1.08 $\times \;10^{-7}$	8.50 $\times \;10^{-9}$		1.11 $\times \;10^{-6}$	3.20 $\times \;10^{-7}$	7.56 $\times \;10^{-8}$	2.95 $\times \;10^{-9}$

**Table A2:** NO${}_{x}$ Coefficients for Positive and Negative Acceleration.

$R_{\mathrm{ij}}^{e}$	For a(t)≥0				$S_{\mathrm{ij}}^{e}$	For a(t)<0			
	j=0	j=1	j=2	j=3		j=0	j=1	j=2	j=3
i=0	−1.08	0.2369	1.47 $\times \;10^{-3}$	−7.82 $\times \;10^{-5}$		−1.08	0.2085	2.19 $\times \;10^{-2}$	8.82 $\times \;10^{-4}$
i=1	1.79 $\times \;10^{-2}$	4.05 $\times \;10^{-2}$	−3.75 $\times \;10^{-3}$	1.05 $\times \;10^{-4}$		2.11 $\times \;10^{-2}$	1.07 $\times \;10^{-2}$	6.55 $\times \;10^{-3}$	6.27 $\times \;10^{-4}$
i=2	2.41 $\times \;10^{-4}$	−4.08 $\times \;10^{-4}$	−1.28 $\times \;10^{-5}$	1.52 $\times \;10^{-6}$		1.63 $\times \;10^{-4}$	−3.23 $\times \;10^{-5}$	−9.43 $\times \;10^{-5}$	−1.01 $\times \;10^{-5}$
i=3	−1.06 $\times \;10^{-6}$	9.42 $\times \;10^{-7}$	1.86 $\times \;10^{-7}$	4.42 $\times \;10^{-9}$		−5.83 $\times \;10^{-7}$	1.83 $\times \;10^{-7}$	4.47 $\times \;10^{-7}$	4.57 $\times \;10^{-8}$

**Table A3:** Fuel Consumption Coefficients for Positive and Negative Acceleration.

$R_{\mathrm{ij}}^{e}$	For a(t)≥0				$S_{\mathrm{ij}}^{e}$	For a(t)<0			
	j=0	j=1	j=2	j=3		j=0	j=1	j=2	j=3
i=0	−7.735	0.2295	−5.61 $\times \;10^{-3}$	9.77 $\times \;10^{-5}$		−7.735	−0.01799	−4.27 $\times \;10^{-3}$	1.88 $\times \;10^{-4}$
i=1	0.02799	0.0068	−7.72 $\times \;10^{-4}$	8.38 $\times \;10^{-6}$		0.02804	7.72 $\times \;10^{-3}$	8.38 $\times \;10^{-4}$	3.39 $\times \;10^{-5}$
i=2	−2.23 $\times \;10^{-4}$	−4.40 $\times \;10^{-5}$	7.90 $\times \;10^{-7}$	8.17 $\times \;10^{-7}$		−2.20 $\times \;10^{-4}$	−5.22 $\times \;10^{-5}$	−7.44 $\times \;10^{-6}$	2.77 $\times \;10^{-7}$
i=3	1.09 $\times \;10^{-6}$	4.80 $\times \;10^{-8}$	3.27 $\times \;10^{-8}$	−7.79 $\times \;10^{-9}$		1.08 $\times \;10^{-6}$	2.47 $\times \;10^{-7}$	4.87 $\times \;10^{-8}$	3.79$\times \;10^{-10}$
